# Intestinal microbiota-associated LysoPC(16:0) promotes endometriosis via PPARγ-mediated M2 macrophage polarization and IL-10 secretion

**DOI:** 10.1016/j.isci.2026.117917

**Published:** 2026-10-15

**Authors:** Na Li, Ming Yuan, Xueyang Jin, Xue Jiao, Shuang Wang, Zhaoyang Zhong, Hongwei Guan, Xu Le, Qianhui Ren, Yu Liu, Xiaoyu Dong, Qiuju Li, Mei Wang, Guoyun Wang

**Affiliations:** 1 Department of Obstetrics and Gynecology, Shandong Provincial Hospital Affiliated to Shandong First Medical University, Jinan, Shandong 250000, China; 2 Gynecology Laboratory, Medical Science and Technology Innovation Center, Shandong First Medical University & Shandong Academy of Medical Sciences, Jinan, Shandong 250000, China; 3 Jinan Key Laboratory of Diagnosis and Treatment of Major Gynaecological Disease, Jinan, Shandong 250000, China; 4 Gynecology Laboratory, Shandong Provincial Hospital, Jinan, Shandong 250000, China; 5 Medical Integration and Practice Center, Cheeloo College of Medicine, Shandong University, Jinan, Shandong 250000, China; 6 Department of Pharmacy, Shandong Provincial Hospital Affiliated to Shandong First Medical University, Jinan, Shandong 250000, China

**Keywords:** endometriosis, gut microbiota, interleukin-10, IL-10, peritoneal lysophosphatidylcholine, LysoPC(16:0), macrophage polarization, PPARγ

## Abstract

Both gut microbiota dysbiosis and altered pelvic immune microenvironment have been linked to endometriosis (EM), but the metabolites connecting the two remain undefined. Combining 16S rDNA sequencing with non-targeted metabolomics in a murine EM model, we found altered gut microbiota composition alongside a marked rise in peritoneal lysophosphatidylcholine (LysoPC(16:0)), linked to the microbiota shift by correlation analysis and fecal transplantation. LysoPC(16:0) promoted M2 macrophage polarization through peroxisome proliferator-activated receptor gamma (PPARγ), an effect observed in two mouse strains. Clodronate-mediated macrophage depletion blocked LysoPC(16:0)-driven lesion growth. LysoPC(16:0) further induced PPARγ-dependent IL-10 transcription, and IL-10 neutralization in vivo reduced lesion weight. Conditioned medium from LysoPC(16:0)-stimulated THP-1 macrophages promoted migration of primary endometrial stromal cells from patients with EM, an effect reversed by GW9662 or IL-10 neutralization. Together, these results outline a gut microbiota-LysoPC(16:0)-PPARγ-M2–IL-10 axis in EM pathogenesis, a mechanistic and therapeutic target for future clinical study.

## Highlights

• Gut microbiota dysbiosis raises peritoneal LysoPC(16:0) in endometriosis
• LysoPC(16:0) drives PPARγ-dependent M2 macrophage polarization and lesion growth
• Macrophage depletion or IL-10 neutralization blocks EM progression *in vivo*
• LysoPC(16:0)-primed macrophages promote migration of human EM stromal cells

## Graphical abstract

**Figure:**
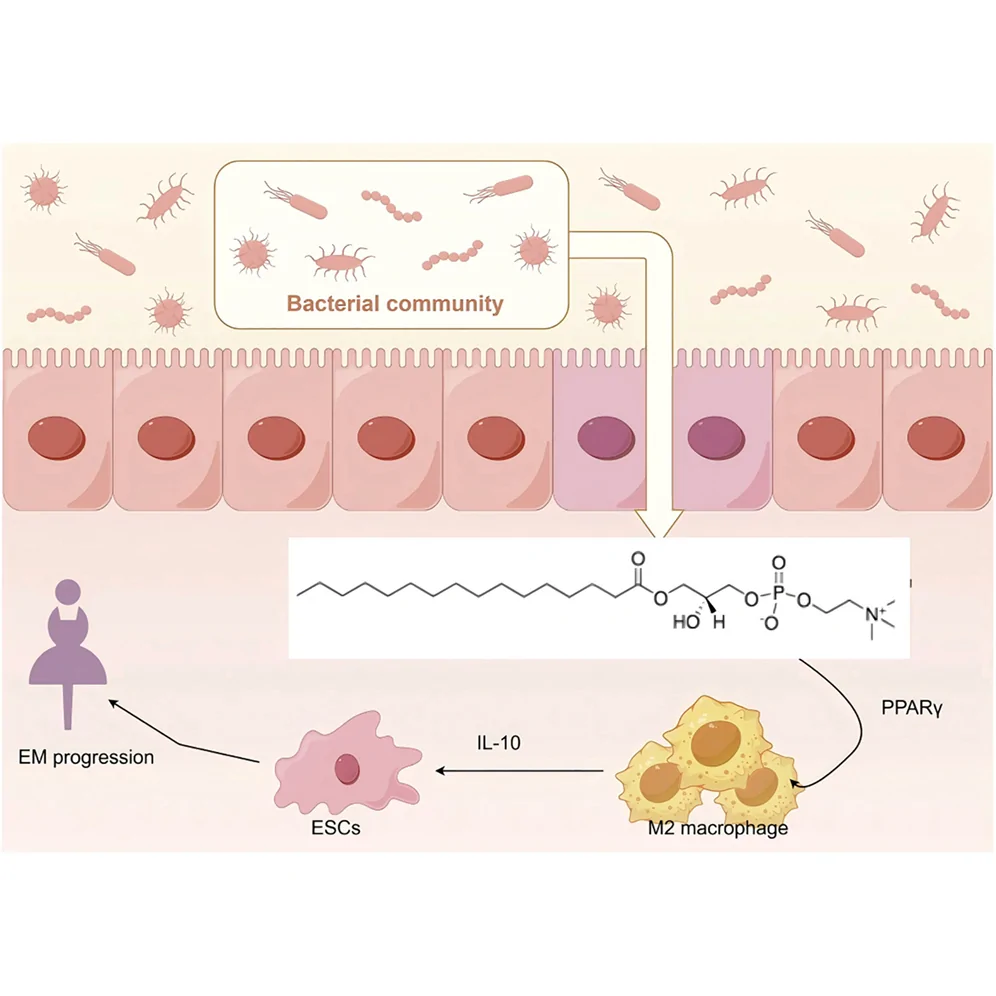

## Teaser

Biological sciences; Immunology; Microbiology

## Introduction

Endometriosis (EM), characterized by the presence of endometrial-like tissue outside the uterine cavity, is a common estrogen-dependent chronic inflammatory disorder affecting over 190 million women of reproductive age globally.^1^ The disease is associated with debilitating symptoms, including pelvic pain, dysmenorrhea, dyspareunia, and infertility, which significantly impair the patients’ quality of life and functioning.^2^ Approximately 50–80% of women with pelvic pain and 50% of those with infertility are affected by EM, underscoring its substantial clinical burden.^3^ Despite extensive investigation, the pathogenesis of EM remains incompletely understood. Leading hypotheses include retrograde menstruation, endometrial stem cell implantation, coelomic metaplasia, and Müllerian duct remnant theories; however, none of these models fully accounts for the complex clinical manifestations of the disease.^4^ Elucidating the mechanisms that drive EM progression therefore remains a priority for developing more effective diagnostic and therapeutic strategies.

Alterations in the peritoneal immune microenvironment are pivotal in EM progression, with the recruitment and polarization of peritoneal macrophages playing a critical role.^5^^,^^6^ Macrophages are broadly categorized into pro-inflammatory M1 and anti-inflammatory M2 subtypes based on their activation state and surface marker expression. In EM, M1 macrophages facilitate the initial colonization of ectopic lesions through pro-inflammatory mediator secretion, whereas M2 macrophages—which express high levels of vascular endothelial growth factor and ARG1, and secrete interleukin-10 (IL-10) and transforming growth factor β (TGF-β)—promote angiogenesis, neurogenesis, and tissue fibrosis, collectively creating a microenvironment that favors lesion survival and growth.^7^^,^^8^ Macrophage polarization states are not fixed; they are continuously shaped by local metabolic conditions and microbial signals.^9^

The gut microbiota, comprising a diverse community of microorganisms residing in the gastrointestinal tract, influences disease progression through its metabolic and immunoregulatory functions, and its maintenance is increasingly recognized as important for female reproductive health.^10^ Several studies have documented gut microbiota alterations in EM. In rhesus monkeys, EM was associated with increased gut inflammation, decreased fecal Bifidobacterium, and expansion of gram-negative bacteria.^11^ In a murine EM model, β-diversity of the fecal microbiota diverged significantly from controls at day 42, with notable shifts in Firmicutes and Bacteroidota abundance.^12^ Comparable dysbiosis has been reported in rat models and in patients with stage 3/4 EM, where reduced microbial diversity and altered Firmicutes/Bacteroidota (F/B) ratios were evident relative to healthy controls.^13^^,^^14^ Although the direction of the F/B ratio change varies across studies—likely reflecting differences in host species, disease stage, diet, and sampling methodology—an abnormal F/B ratio is broadly recognized as a marker of gut microbiota dysbiosis. Despite these observations, the specific microbial metabolites that translate gut dysbiosis into pelvic immune remodeling remain largely uncharacterized. The gut microbiota exerts remote effects on host physiology through the production of bioactive metabolites, including short-chain fatty acids, bile acids, and tryptophan derivatives, forming the basis of axes such as the gut-liver and gut-brain axes that have been extensively studied in inflammatory bowel disease, obesity, diabetes, and neurodegenerative disorders.^15^ The gut-uterus axis has more recently attracted attention as a mechanistic framework for understanding microbiota-reproductive health interactions.^10^ Among gut-associated lipid metabolites, peritoneal lysophosphatidylcholine (LysoPC(16:0))—generated primarily through phospholipase A2 (PLA2)-mediated hydrolysis of membrane phospholipids—has been identified as a circulating biomarker in rheumatoid arthritis and systemic lupus erythematosus,^16^^,^^17^ and has recently been shown to modulate macrophage function in other inflammatory contexts.^18^ Whether LysoPC(16:0) plays a role in EM has not previously been examined. Notably, lysophospholipids and structurally related fatty-acid derivatives can act as endogenous ligands for peroxisome proliferator-activated receptor gamma (PPARγ), a nuclear receptor that governs macrophage polarization programs,^19^ raising the possibility that LysoPC(16:0) may influence macrophage phenotype through this pathway—a link that has not previously been explored in the context of EM.

Although prior studies have highlighted associations between the microbiota, peritoneal immunity, and EM, the specific metabolites that mediate these relationships and their downstream mechanisms remain unclear. The present study therefore employed 16S rDNA sequencing, non-targeted LC-MS metabolomics, and a series of *in vivo* and *in vitro* approaches to systematically investigate gut microbiota-metabolite interactions in EM mice. To strengthen mechanistic and translational evidence, experiments were additionally conducted in two mouse strains (C57BL/6N and BALB/c), with macrophage depletion by clodronate liposomes, *in vivo* IL-10 neutralization, and human primary ESC models derived from patients with confirmed EM. These analyses identified LysoPC(16:0) as a gut microbiota-associated metabolite that drives M2 macrophage polarization via PPARγ and promotes IL-10-dependent ectopic lesion progression, implicating the “gut microbiota-LysoPC(16:0)–PPARγ–M2–IL-10” axis as a mechanistic and therapeutic target in EM. As the present work is primarily based on murine models and cell-based human systems, its findings should be interpreted as preclinical mechanistic evidence, with clinical applicability to be established in future patient studies.

## Results

### Gut microbiota composition is altered in mice with EM

The fecal microbiome of EM and control mice was profiled by 16S rDNA sequencing during diestrus at day 42 post-model establishment (Figures 1A and 1B). After quality filtering, 700 amplicon sequence variants (ASVs) were shared between groups, with 583 unique to controls and 536 unique to EM mice (Figure 1C). Alpha-diversity indices—including Chao1, Shannon, Pielou evenness, and Goods coverage—did not differ significantly between groups (Figures 1D–1G), whereas beta-diversity analysis revealed clear structural separation by unweighted UniFrac-based PCoA, hierarchical clustering, and ANOSIM (Figures 1H–1J). At the phylum level, EM mice showed reduced Firmicutes abundance and increased Bacteroidota, with a significantly altered F/B ratio compared to controls (Figures 1K and 1L). LEfSe analysis further delineated taxon-level compositional differences between groups (Figure 1M), and indicator species analysis nominated *g_Ruminococcus, g_Coprococcus,* g_*Muribaculum*, and *g_Duncaniella* as candidate EM-associated biomarkers (Figure 1N). KEGG-based functional prediction identified enrichment of sphingolipid biosynthesis and metabolism pathways in EM mice (Figure 1O).

**Figure 1:**
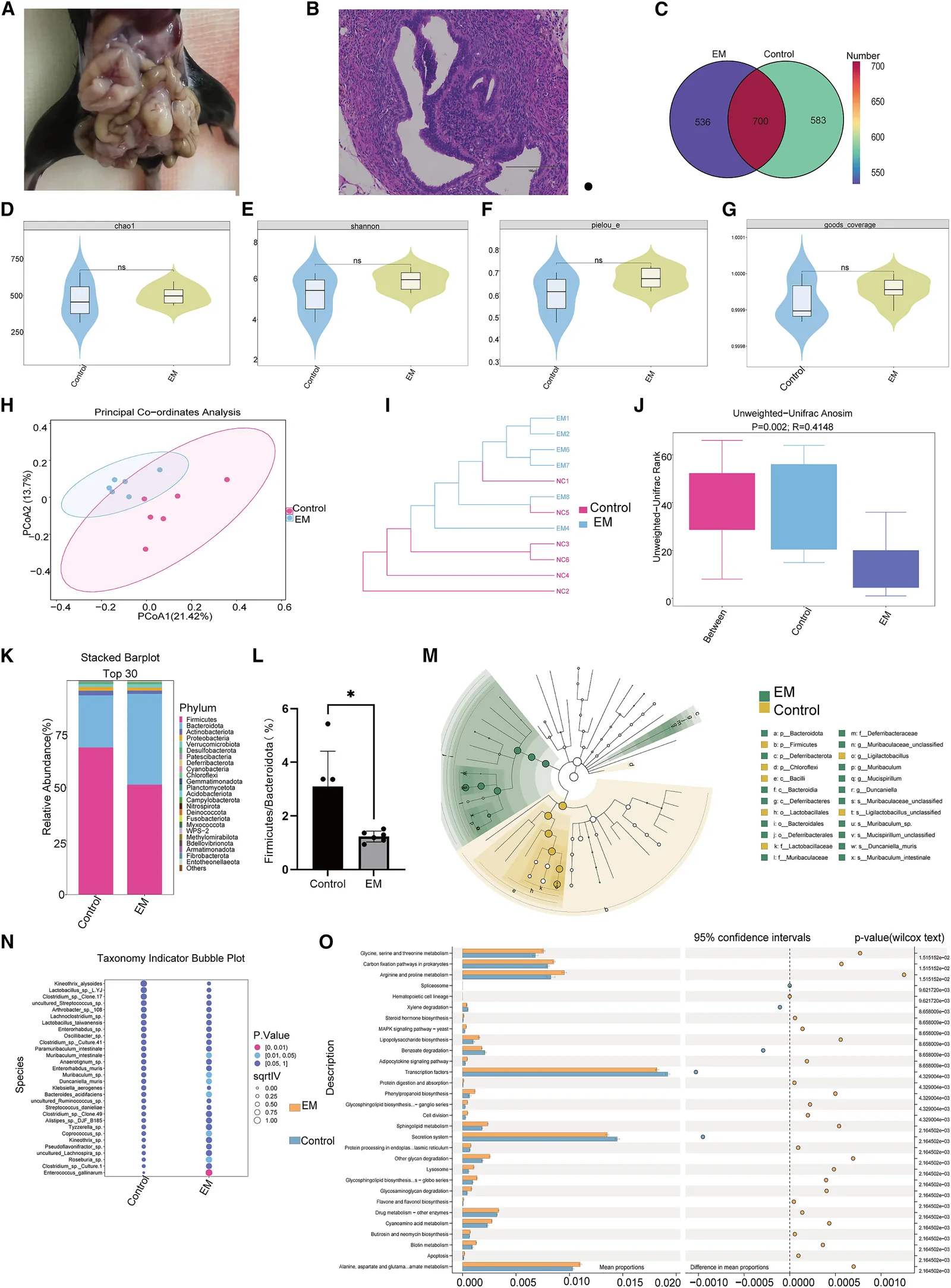
Gut microbiota structure is altered in mice with endometriosis (A) Representative lesion images at day 42, photographed alongside a graduated scale board (cm/mm divisions). (B) Hematoxylin and eosin staining of lesion tissue (10×); scale bars, 150 μm. (C) Venn diagram of unique and shared ASVs between EM and control groups. (D–G) Alpha diversity indices (Chao1, Shannon, Pielou’s evenness, Good’s coverage); Wilcoxon rank-sum test. Violin plots show the kernel density distribution of the data; embedded boxplots: box bounds, 25th and 75th percentiles (interquartile range, IQR); center line, median; whiskers, the most extreme values within 1.5 × IQR; points beyond the whiskers, outliers. ns, not significant. (H) Principal coordinates analysis (PCoA) based on unweighted UniFrac distance. (I) Hierarchical clustering (UPGMA) based on unweighted UniFrac distance. (J) Analysis of similarities (ANOSIM) based on unweighted UniFrac distance, a non-parametric permutation test; R and *p* values are indicated on the plot. The x axis shows the distribution of between-group pairwise distances (Between) and of within-group pairwise distances for each group; the y axis shows the rank of these distances. Boxplots: box bounds, 25th and 75th percentiles; center line, median; whiskers, minimum and maximum values (no data point lies beyond 1.5 × IQR of the box bounds). (K) Stacked bar plot of the relative abundance of the top 30 phyla. (L) Comparison of the Firmicutes/Bacteroidota (F/B) ratio; unpaired Welch’s *t* test. (M) LEfSe cladogram of differentially abundant taxa, identified by the Kruskal-Wallis rank-sum test followed by pairwise Wilcoxon rank-sum test and linear discriminant analysis (LDA). (N) Indicator species analysis of the top 30 genera; dot size represents the square root of the indicator value (IndVal), and dot color represents the permutation-test *p* value. (O) KEGG-based differential functional analysis predicted with PICRUSt2 and visualized with STAMP; two-sided Wilcoxon rank-sum test; the top 30 functions with *p* < 0.05 are shown. Left, mean proportion of each functional category in the EM and control groups; right, difference in mean proportions between groups shown with 95% confidence intervals, with the corresponding *p* values listed alongside. Unless otherwise indicated, *n* = 6 mice per group. ∗*p* < 0.05, ∗∗*p* < 0.01, and ∗∗∗*p* < 0.001. Data in (L) are represented as mean ± SD.

### LysoPC(16:0) is elevated in the peritoneal lavage fluid of EM mice

Non-targeted metabolomics of peritoneal lavage fluid was performed following quality control processing with metaX. Partial least-squares discriminant analysis (PLS-DA) revealed clear separation between EM and control metabolic profiles (Figure 2A), and a 200-permutation test confirmed model robustness without overfitting (Figure 2B). Applying thresholds of FC ≥ 1.2 or FC ≤ 1/1.2, *p* < 0.05 (*t* test), and VIP ≥1 identified 26 upregulated and 2 downregulated metabolites in the EM group (Figure 2D). Among these, LysoPC(16:0) exhibited one of the largest fold-changes, as highlighted in a heatmap of the top 28 differential metabolites (Figures 2C and 2E). KEGG pathway enrichment of the full differential metabolite set identified glycerophospholipid metabolism, choline metabolism, linoleic acid metabolism, and unsaturated fatty acid biosynthesis among the top 20 enriched pathways (Figure 2F).

**Figure 2:**
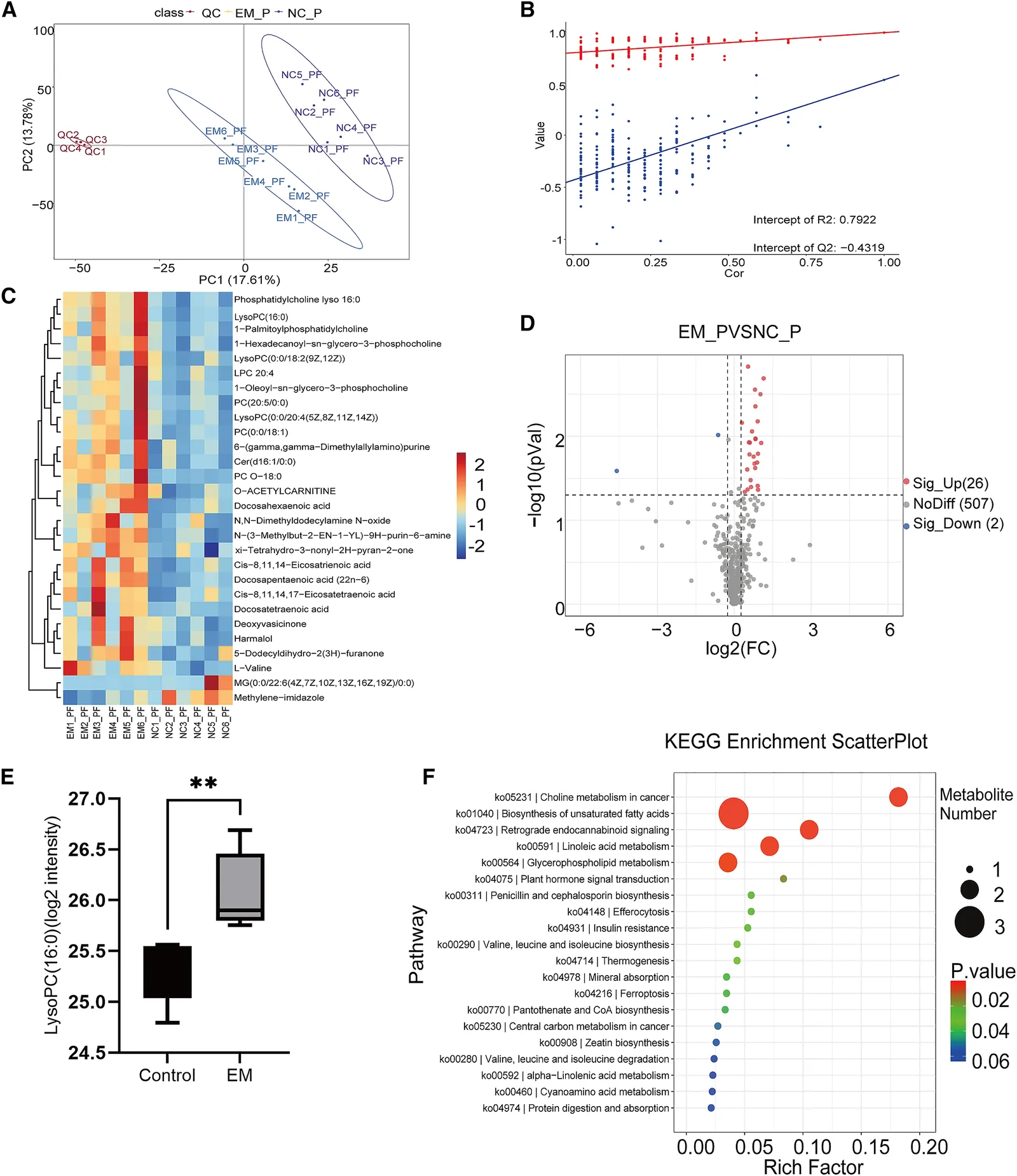
LysoPC(16:0) is elevated in the peritoneal lavage fluid of EM mice (A) Partial least squares-discriminant analysis (PLS-DA) score plot of peritoneal metabolomes from EM and control mice. (B) Two-hundred-permutation validation of the PLS-DA model. (C) Heatmap of the top 28 differential metabolites; two independently annotated features corresponding to lysophosphatidylcholine 16:0 are shown as separate rows. (D) Volcano plot of differential metabolites (fold change ≥1.2 or ≤1/1.2, VIP ≥1, *p* < 0.05). (E) Log2 intensity of LysoPC(16:0) in control and EM groups; unpaired Student’s *t* test. Boxplots: box bounds, 25th and 75th percentiles; center line, median; whiskers, minimum and maximum values. (F) KEGG pathway enrichment scatterplot of the top 20 pathways ranked by *p* value; dot size represents the number of matched metabolites. *n* = 6 mice per group; ∗*p* < 0.05, ∗∗*p* < 0.01, and ∗∗∗*p* < 0.001.

### FMT from EM donors elevates peritoneal LysoPC(16:0) and promotes M2 macrophage polarization

To explore whether the altered gut microbiota could account for elevated peritoneal LysoPC(16:0), Spearman’s correlation analysis was performed between significantly different genera and differential metabolites (rho ≥0.8, *p* < 0.05). Multiple genera—including *g_Muribaculum, g_Mucispirillum, g_Ruminococcus*, and *g_Lactiplantibacillus*—showed significant positive correlations with LysoPC(16:0) (Figure 3A), suggesting a potential microbiota-metabolite link. To test this causally, mice were rendered pseudo-germ-free by a seven-day antibiotic cocktail (ABX) regimen before model establishment and subsequently gavaged with PBS (FMT-PBS), control-donor feces (FMT-NC), or EM-donor feces (FMT-EM) every three days (Figure 3B). Peritoneal LysoPC(16:0) was significantly higher in FMT-EM recipients than in either control group (Figure 3D). FMT-EM mice also developed heavier lesions (Figures 3C–3E and 3F) and showed a greater proportion of CD206+ macrophages in peritoneal lavage fluid compared with FMT-NC and FMT-PBS mice (Figures 3G and 3H). These results indicate that EM-associated gut microbiota is sufficient to elevate peritoneal LysoPC(16:0) and promote peritoneal M2 macrophage accumulation, thereby facilitating lesion growth.

**Figure 3:**
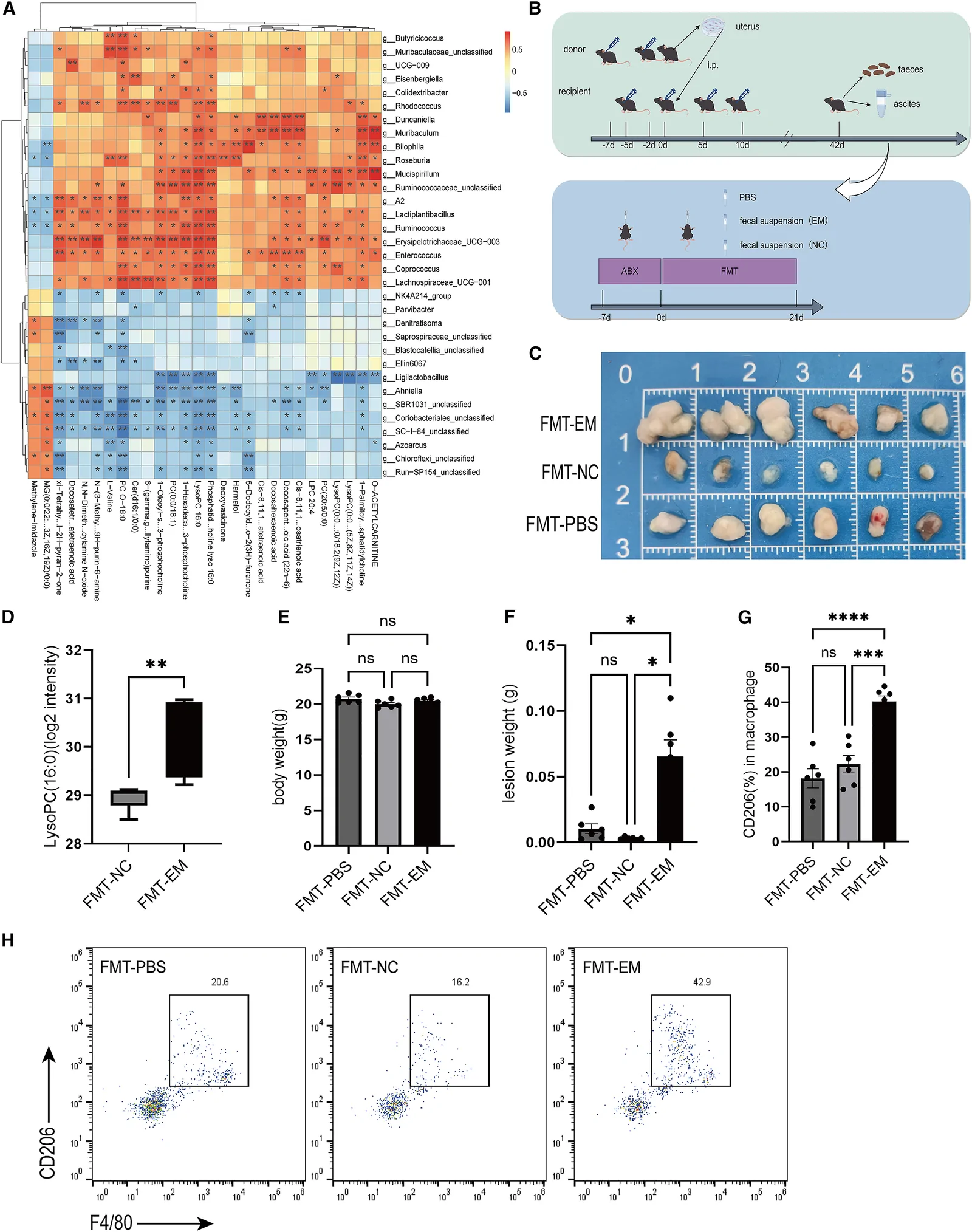
FMT from EM donors regulates peritoneal LysoPC(16:0) and M2 macrophage polarization (A) Spearman correlation heatmap between significantly altered genera and differential metabolites (rho ≥0.8, *p* < 0.05). (B) Experimental flowchart for antibiotic pretreatment and FMT. (C) Representative lesion photographs post-FMT, photographed alongside a graduated scale board (cm/mm divisions). (D) Log2 intensity of LysoPC(16:0) in peritoneal lavage fluid of FMT-NC and FMT-EM mice; Mann-Whitney test. Boxplots: box bounds, 25th and 75th percentiles; center line, median; whiskers, minimum and maximum values. (E) Body weight across FMT-PBS, FMT-NC, and FMT-EM groups; ordinary one-way ANOVA with Tukey’s post hoc test. (F) Lesion weight across groups; Brown-Forsythe and Welch ANOVA with Dunnett’s T3 multiple comparisons test. (G) Proportion of CD206+ macrophages in peritoneal lavage fluid across groups; Brown-Forsythe and Welch ANOVA with Dunnett’s T3 multiple comparisons test. (H) Representative flow cytometry plots of peritoneal macrophages. *n* = 6 mice per group; ∗*p* < 0.05, ∗∗*p* < 0.01, and ∗∗∗*p* < 0.001; ns, not significant. Data in (E–G) are represented as mean ± SEM.

### LysoPC(16:0) promotes M2 polarization of BMDMs and enhances MESC migration

Bone marrow-derived macrophages (BMDMs) were matured with M-CSF for one week and then allocated to NC, IL-4, and IL-4 + LysoPC(16:0) groups. After 24 h of stimulation, flow cytometry showed that 50 μM LysoPC(16:0) significantly augmented IL-4-induced CD206 expression (Figures 4A–4C). In a Transwell co-culture assay, MESCs placed in the upper chamber migrated significantly more when co-cultured with IL-4 + LysoPC(16:0)-stimulated BMDMs in the lower chamber than with NC or IL-4-alone controls (Figures 4D–4F). Additionally, a phagocytosis assay using PE-conjugated microspheres demonstrated that LysoPC(16:0)-treated BMDMs exhibited enhanced particle uptake over 2 h (Figures 4G and 4H), consistent with prior reports that LysoPC promotes macrophage phagocytic activity.

**Figure 4:**
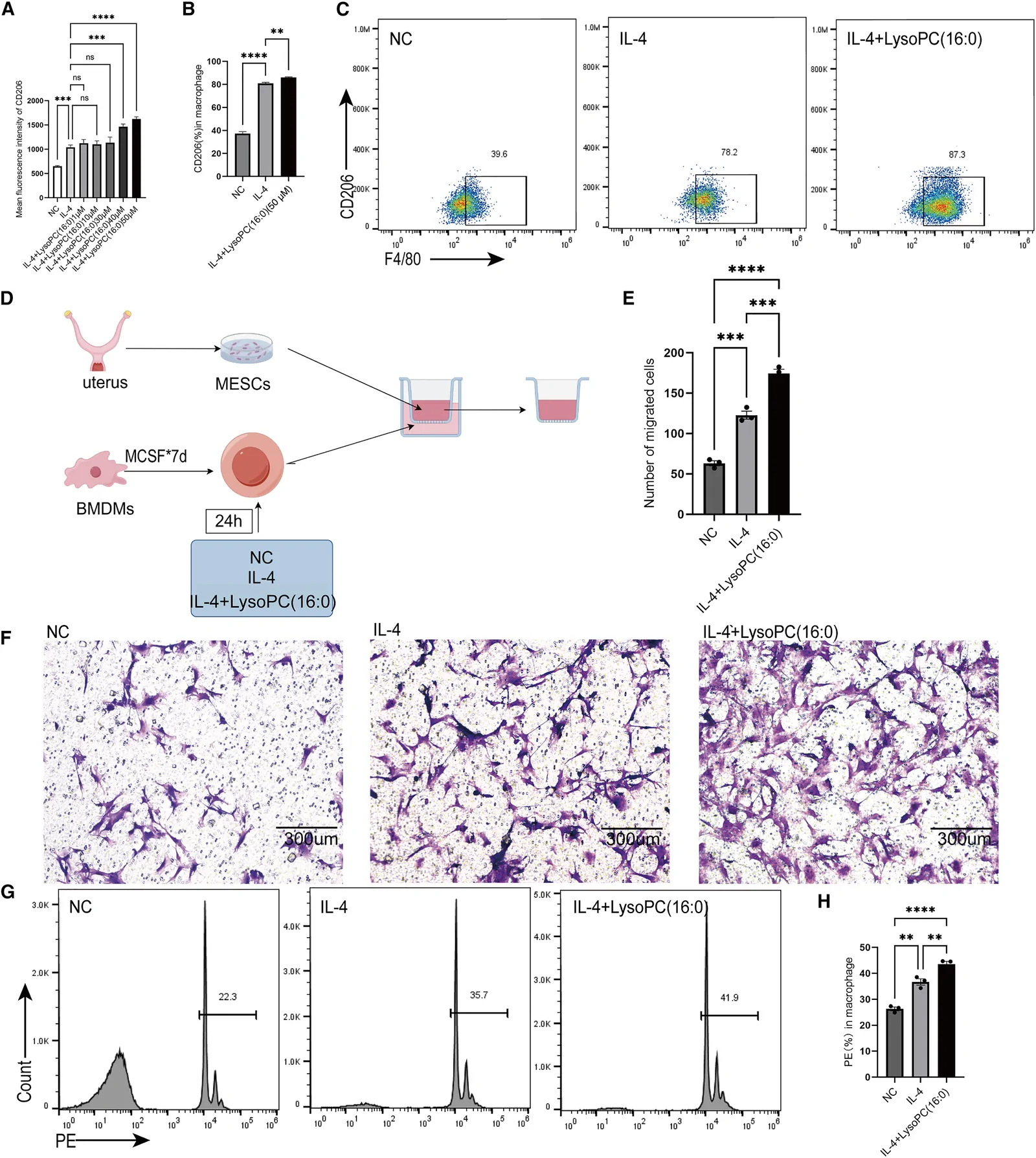
LysoPC(16:0) promotes M2 polarization of BMDMs and enhances their pro-migratory effect on endometrial stromal cells (A) Mean fluorescence intensity of CD206 in BMDMs stimulated with NC, IL-4, or IL-4 plus LysoPC(16:0) at 1, 10, 30, 40, or 50 μM; Brown-Forsythe and Welch ANOVA with Dunnett’s T3 multiple comparisons test against the IL-4 group. (B) Proportion of CD206+ BMDMs under NC, IL-4, and IL-4 + LysoPC(16:0) (50 μM) conditions; Brown-Forsythe and Welch ANOVA with Dunnett’s T3 multiple comparisons test against the IL-4 group. (C) Representative flow cytometry plots of CD206+ BMDMs under NC, IL-4, and IL-4 + LysoPC(16:0) conditions. (D) Schematic of the Transwell co-culture assay: BMDMs were stimulated with NC, IL-4, or IL-4 + LysoPC(16:0) for 24 h before co-culture with mouse endometrial stromal cells (MESCs). (E) Number of migrated MESCs; ordinary one-way ANOVA with Tukey’s post hoc test. (F) Representative images of migrated MESCs stained with crystal violet; scale bars, 300 μm. (G) Representative flow cytometry histograms of PE-labeled microsphere uptake by BMDMs. (H) Proportion of PE + BMDMs; ordinary one-way ANOVA with Tukey’s post hoc test. *n* = 2 technical replicates from 3 biological replicates (A, B); *n* = 3 biological replicates (E and H); ∗*p* < 0.05, ∗∗*p* < 0.01, ∗∗∗*p* < 0.001, and ∗∗∗∗*p* < 0.0001; ns, not significant. Data are represented as mean ± SEM.

### LysoPC(16:0) promotes endometriotic lesion growth and peritoneal M2 macrophage accumulation *in vivo*

Following model establishment, C57BL/6N mice were allocated to three groups: EM vehicle control, AACOCF3 (PLA2 inhibitor), and LysoPC(16:0) (Figure 5A). AACOCF3 was used to suppress endogenous LysoPC(16:0) production by inhibiting PLA2-mediated phospholipid hydrolysis. After two weeks of daily intraperitoneal injection, body weights did not differ significantly among groups. Mice treated with LysoPC(16:0) developed larger lesions than EM vehicle controls, whereas AACOCF3-treated mice showed smaller lesions than the EM group (Figures 5B and 5C). Flow cytometry of peritoneal macrophages showed a higher proportion of CD206+ cells in the LysoPC(16:0) group and a lower proportion in the AACOCF3 group relative to EM controls (Figures 5E and 5F). These results indicate that LysoPC(16:0) promotes peritoneal M2 macrophage accumulation and ectopic lesion growth, and that suppressing its endogenous production has the opposite effect.

**Figure 5:**
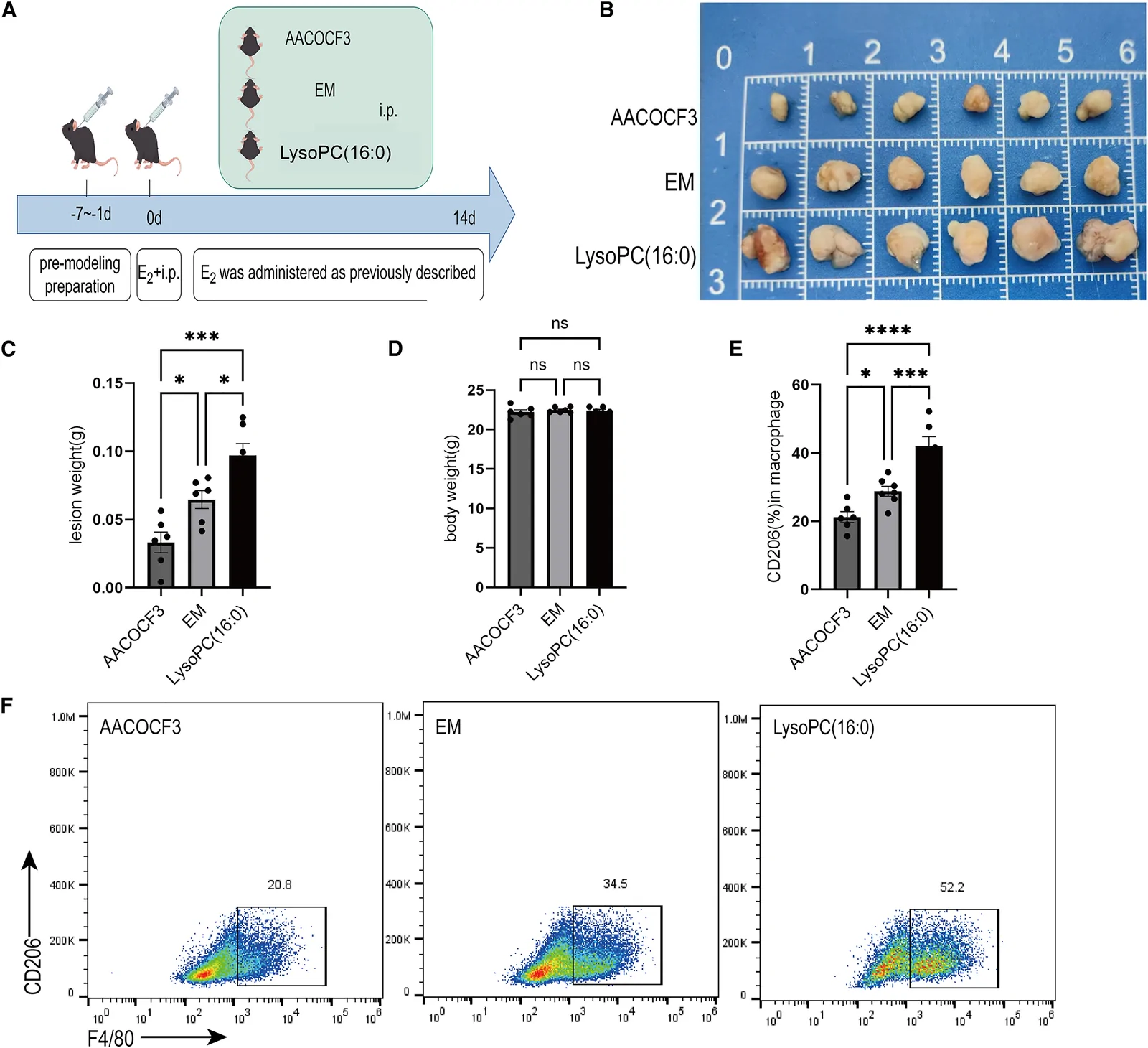
LysoPC(16:0) promotes endometriotic lesion growth and peritoneal M2 macrophage accumulation (A) Experimental flowchart: C57BL/6N EM mice were allocated to three groups (EM vehicle, AACOCF3, LysoPC(16:0)) and received daily intraperitoneal injections for two weeks. (B) Representative lesion photographs across groups, photographed alongside a graduated scale board (cm/mm divisions). (C) Lesion weight; ordinary one-way ANOVA with Tukey’s post hoc test. (D) Body weight; ordinary one-way ANOVA with Tukey’s post hoc test. (E) Proportion of CD206+ macrophages in peritoneal lavage fluid; ordinary one-way ANOVA with Tukey’s post hoc test. (F) Representative flow cytometry plots of CD206+ macrophages in peritoneal lavage fluid. *n* = 6 mice per group; ∗*p* < 0.05, ∗∗*p* < 0.01, and ∗∗∗*p* < 0.001; ns, not significant. Data are represented as mean ± SEM.

### PPARγ mediates the M2-polarizing and pro-endometriotic effects of LysoPC(16:0)

To determine whether PPARγ signaling underlies the M2-polarizing activity of LysoPC(16:0), BMDMs were stimulated for 24 h under four conditions: NC, IL-4, IL-4 + LysoPC(16:0), and IL-4 + LysoPC(16:0) + GW9662, with GW9662 applied 4 h before co-stimulation. LysoPC(16:0) significantly augmented IL-4-induced CD206+ polarization, and this was substantially reduced by GW9662 (Figures 6A and 6B), establishing PPARγ as necessary for the M2-polarizing effect of LysoPC(16:0) *in vitro*.

**Figure 6:**
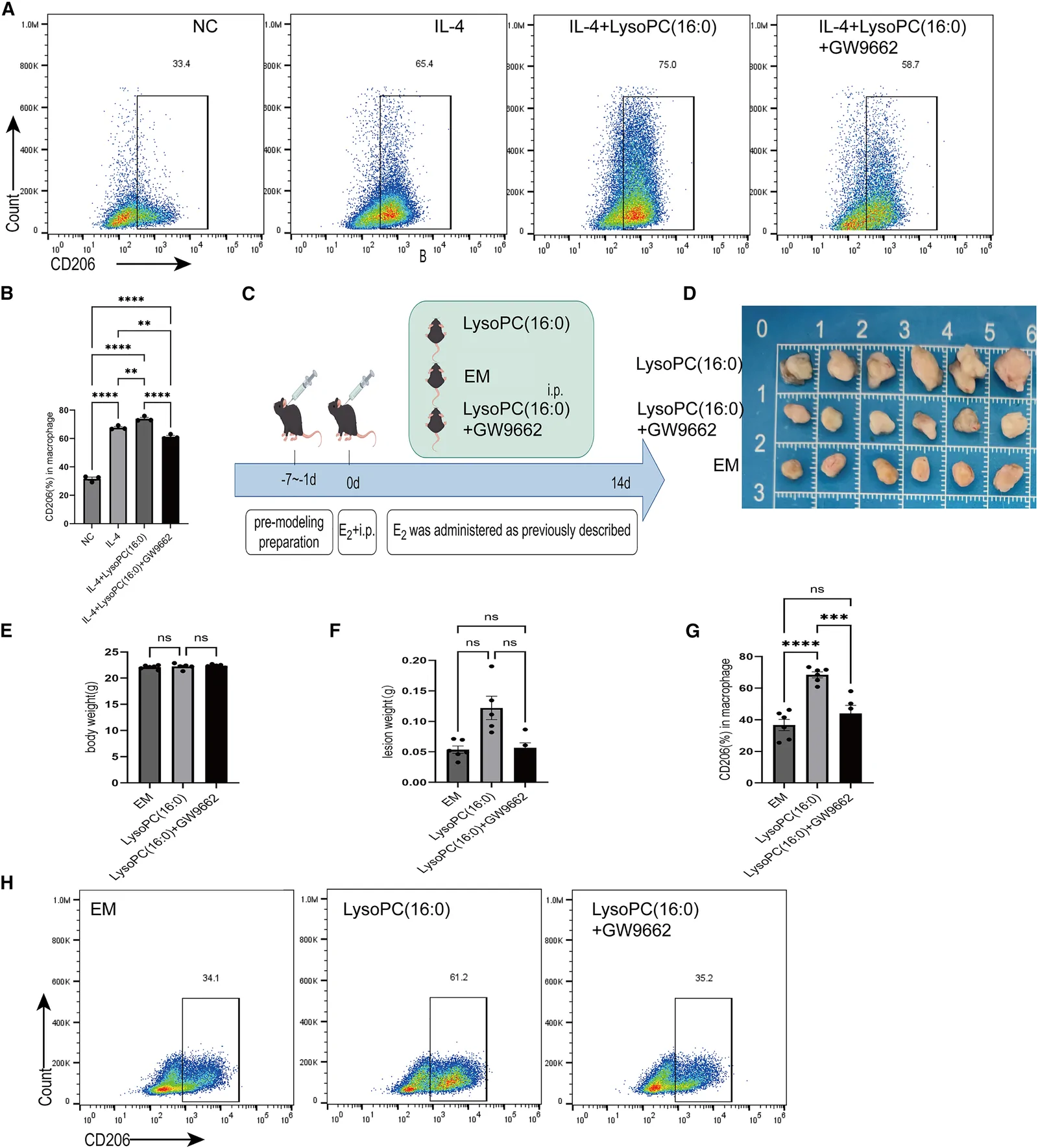
PPARγ mediates LysoPC(16:0)-induced M2 polarization and lesion growth (A) Representative flow cytometry plots of CD206+ BMDMs under four conditions (NC, IL-4, IL-4 + LysoPC(16:0), and IL-4 + LysoPC(16:0) + GW9662). (B) Proportion of CD206+ cells; ordinary one-way ANOVA with Dunnett’s multiple comparisons test against the IL-4 group. (C) Experimental flowchart: C57BL/6N EM mice were allocated to three groups (EM vehicle, LysoPC(16:0), and LysoPC(16:0) + GW9662). (D) Representative lesion photographs, photographed alongside a graduated scale board (cm/mm divisions). (E) Body weight; ordinary one-way ANOVA with Tukey’s post hoc test. (F) Lesion weight; Brown-Forsythe and Welch ANOVA with Dunnett’s T3 multiple comparisons test. (G) Proportion of peritoneal CD206+ macrophages; ordinary one-way ANOVA with Tukey’s post hoc test. (H) Representative flow cytometry plots of peritoneal CD206+ macrophages (*in vivo* validation). *n* = 3 biological replicates (A and B); *n* = 6 mice per group (D–H); ∗*p* < 0.05, ∗∗*p* < 0.01, ∗∗∗*p* < 0.001, and ∗∗∗∗*p* < 0.0001; ns, not significant. Data are represented as mean ± SEM.

To confirm this *in vivo*, a separate cohort of C57BL/6N EM mice was allocated to three groups: EM vehicle, LysoPC(16:0), and LysoPC(16:0) + GW9662 (Figure 6C). At two weeks, body weights were comparable across groups. LysoPC(16:0)-treated mice developed heavier lesions and showed a higher proportion of peritoneal CD206+ macrophages than EM vehicle controls; GW9662 co-administration significantly reduced both parameters compared with the LysoPC(16:0) group (Figures 6D–6F, 6G, and 6H). To assess whether these findings extend to a second mouse strain, BALB/c EM mice were allocated to four groups in a single experiment: EM vehicle, AACOCF3, LysoPC(16:0), and LysoPC(16:0) + GW9662. The directional results were consistent with those in C57BL/6N mice across all outcome measures (Figure S1).

### Peritoneal macrophages and macrophage-derived IL-10 are required for LysoPC(16:0)-driven EM progression

The above data showed that LysoPC(16:0) drives M2 polarization and lesion growth through PPARγ, but left open whether peritoneal macrophages are causally required for these effects. To address this directly, C57BL/6N EM mice receiving LysoPC(16:0) were co-administered clodronate liposomes (CL) to deplete peritoneal macrophages, or empty control liposomes (EL), intraperitoneally beginning on day 1 post-model establishment and repeated every 3 days. At day 14, CL-treated mice showed markedly fewer F4/80^+^ cells in peritoneal lavage fluid and significantly lower lesion weights than EL controls, despite equivalent LysoPC(16:0) exposure (Figures 7A–7D). Macrophage depletion therefore abolished the pro-endometriotic effect of LysoPC(16:0), demonstrating that peritoneal macrophages are required mediators rather than incidental bystanders. We next asked which secreted molecule accounts for the lesion-promoting activity of LysoPC(16:0)-polarized M2 macrophages. RT-qPCR of BMDMs stimulated under four conditions—NC, IL-4, IL-4 + LysoPC(16:0), and IL-4 + LysoPC(16:0) + GW9662—showed that LysoPC(16:0) significantly upregulated *Il10* mRNA in IL-4-primed cells, and this induction was abolished by GW9662 (Figure 7E), placing *Il10* transcription downstream of PPARγ in LysoPC(16:0)-stimulated macrophages.

**Figure 7:**
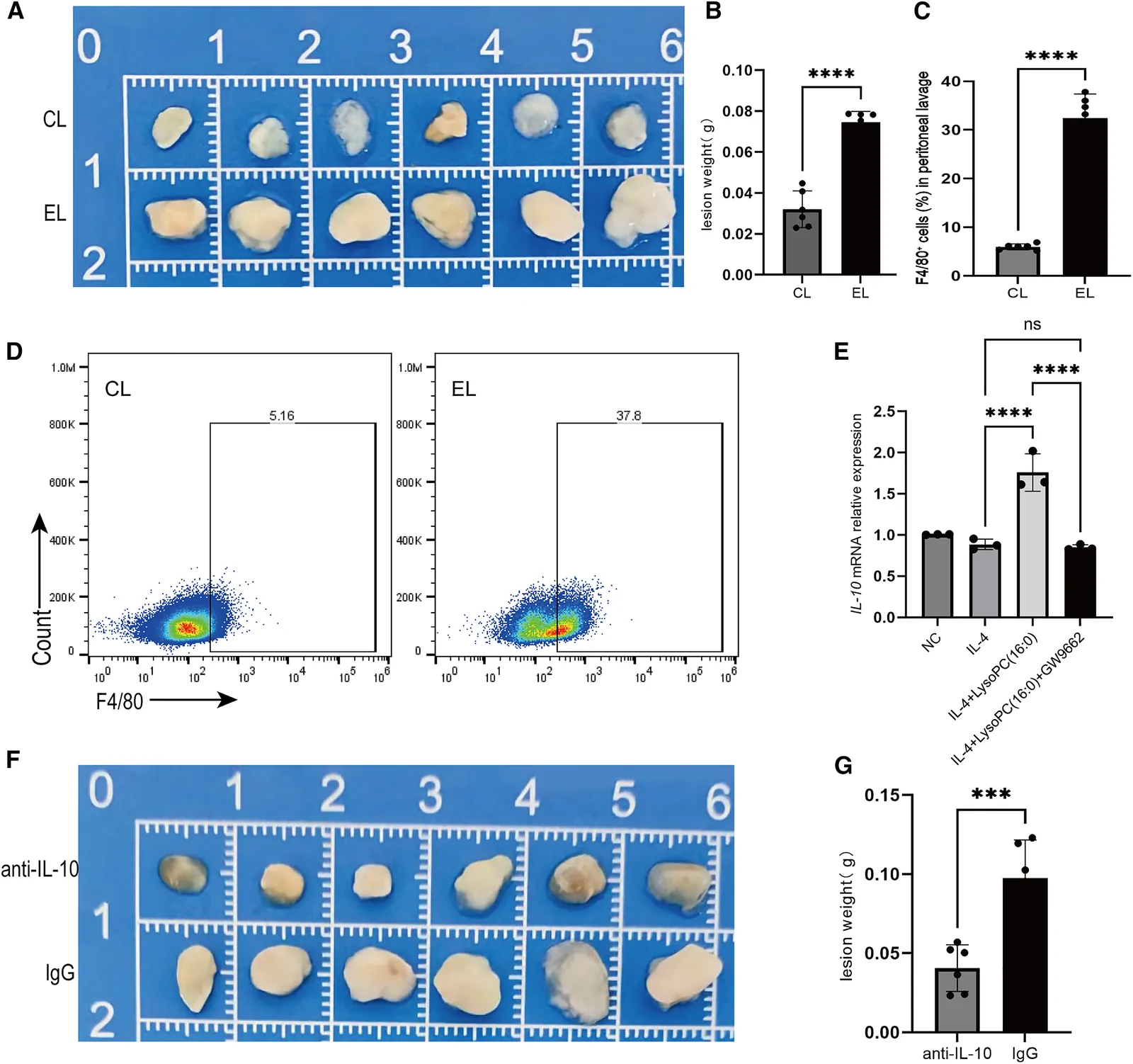
Peritoneal macrophages and IL-10 are required for LysoPC(16:0)-driven EM progression (A) Representative lesion photographs from CL- and EL-treated mice, photographed alongside a graduated scale board (cm/mm divisions). (B) Lesion weight; unpaired Student’s *t* test. (C) Proportion of F4/80^+^ cells in peritoneal lavage fluid; unpaired Welch’s *t* test. (D) Representative flow cytometry plots of F4/80^+^ cells in peritoneal lavage fluid from CL- and EL-treated mice. (E) RT-qPCR of *Il10* mRNA in BMDMs stimulated with NC, IL-4, IL-4 + LysoPC(16:0), or IL-4 + LysoPC(16:0) + GW9662; ordinary one-way ANOVA with Šídák’s multiple comparisons test among the IL-4, IL-4 + LysoPC(16:0), and IL-4 + LysoPC(16:0) + GW9662 groups. (F) Representative lesion photographs from anti-IL-10- and isotype control-treated mice, photographed alongside a graduated scale board (cm/mm divisions). (G) Lesion weight; unpaired Student’s *t* test. *n* = 6 mice per group (A–D, F, and G); *n* = 3 technical replicates from 3 biological replicates (E); ∗*p* < 0.05, ∗∗*p* < 0.01, ∗∗∗*p* < 0.001, and ∗∗∗∗*p* < 0.0001; ns, not significant. Data are represented as mean ± SD (B, E, and G) and mean ± SEM (C).

To test whether IL-10 is functionally required *in vivo*, EM mice receiving LysoPC(16:0) were co-treated with a neutralizing anti-IL-10 antibody (200 μg per mouse, every 3 days) or an isotype control for two weeks. Anti-IL-10-treated mice developed significantly smaller lesions than isotype controls (Figures 7F and 7G).

### The LysoPC(16:0)-PPARγ-M2-IL-10 axis promotes migration of human primary ESCs

To determine whether the LysoPC(16:0)-PPARγ-M2 relationship holds in human macrophages, THP-1 monocytes were differentiated into macrophages by PMA treatment, rested for 24 h, and then stimulated under four conditions: NC, IL-4/IL-13, IL-4/IL-13 + LysoPC(16:0), and IL-4/IL-13 + LysoPC(16:0) + GW9662. LysoPC(16:0) significantly increased the proportion of CD206^+^ cells compared with IL-4/IL-13 alone, and GW9662 reversed this effect (Figures 8A and 8B).

**Figure 8:**
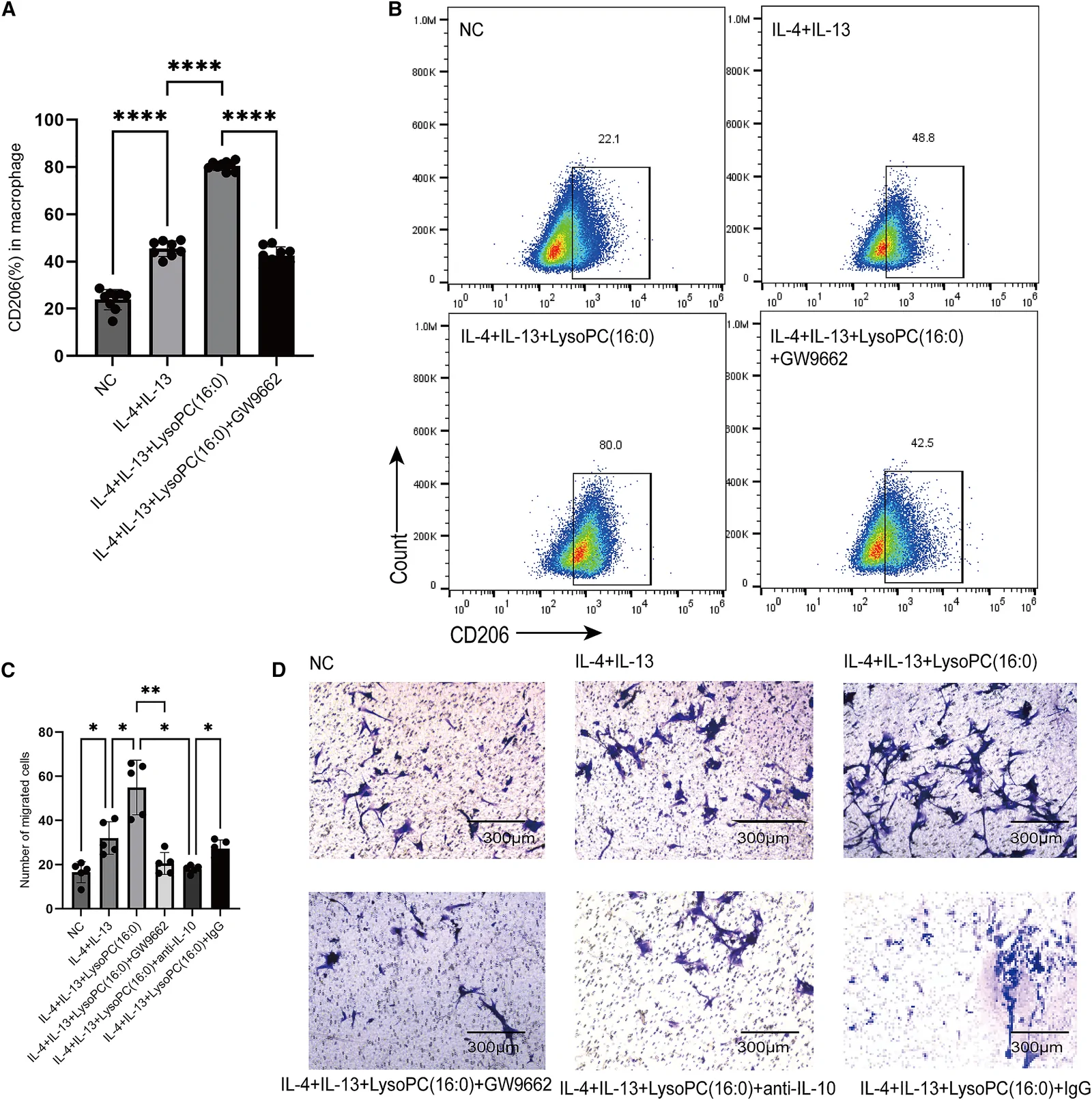
The LysoPC(16:0)-PPARγ-M2-IL-10 axis promotes migration of human primary ESCs (A) Proportion of CD206^+^ cells in THP-1-derived macrophages stimulated under four conditions (NC, IL-4/IL-13, IL-4/IL-13 + LysoPC(16:0), and IL-4/IL-13 + LysoPC(16:0) + GW9662); ordinary one-way ANOVA with Šídák’s multiple comparisons test among sequential conditions. (B) Representative flow cytometry plots of CD206^+^ THP-1-derived macrophages under the four stimulation conditions. (C) Quantification of migrated ESCs from five patients under each of six conditioned medium conditions; ordinary one-way ANOVA with Šídák’s multiple comparisons test against the NC group. (D) Representative crystal violet-stained migration images of human primary ESCs exposed to conditioned medium from THP-1 macrophages under six stimulation conditions; scale bars, 300 μm. *n* = 3 technical replicates from 3 biological replicates (A); *n* = 5 patients (C); ∗*p* < 0.05, ∗∗*p* < 0.01, and ∗∗∗∗*p* < 0.0001. Data are represented as mean ± SD.

Conditioned medium (CM) was then collected from THP-1 macrophages stimulated under six conditions: NC, IL-4/IL-13, IL-4/IL-13 + LysoPC(16:0), IL-4/IL-13 + LysoPC(16:0) + GW9662, IL-4/IL-13 + LysoPC(16:0) + anti-IL-10 antibody, and IL-4/IL-13 + LysoPC(16:0) + isotype control. Primary ESCs were isolated from ectopic lesions of five patients with surgically confirmed EM and exposed to each CM in Transwell migration assays. CM from LysoPC(16:0)-stimulated macrophages promoted significantly greater ESC migration than CM from IL-4/IL-13-alone conditions across all five patient samples. This enhancement was substantially reduced by GW9662 or anti-IL-10 treatment, but not by the isotype control (Figures 8C and 8D).

## Discussion

In this study, disrupted gut microbiota composition was observed in EM mice, and fecal microbiota transplantation experiments demonstrated that EM-associated microbiota elevates peritoneal LysoPC(16:0) levels. LysoPC(16:0) in turn promoted M2 polarization of peritoneal macrophages through PPARγ, with IL-10 identified as the key downstream effector mediating lesion growth. These findings were validated across two mouse strains and in human primary cells, highlighting the “gut microbiota–LysoPC(16:0)–PPARγ–M2–IL-10” axis as a mechanistic framework for understanding EM pathogenesis.

The intestinal microbiota, a substantial “microbial organ” of the human body, influences host physiological and pathological processes through alterations in metabolite production.^20^ The present study revealed notable structural changes in the gut microbiota of EM mice, consistent with prior work linking intestinal flora imbalances to disorders of the female reproductive system.^21^ Altered microbial diversity and abnormal species enrichment have been documented in polycystic ovary syndrome and EM,^22^^,^^23^ suggesting that the gut microbiota may influence the pelvic microenvironment via the “gut–uterus” axis, thereby affecting reproductive function.^10^ Regarding the F/B ratio, the present data showed decreased Firmicutes and increased Bacteroidota in EM mice; however, the direction of this shift has been variable across studies, with some reporting a higher F/B ratio in EM groups,^12^ others the opposite,^24^ and some detecting no significant differences.^25^ This variability likely reflects differences in host species, estrous cycle stage, diet, and housing conditions rather than a fundamental inconsistency in the biology. Regardless of directionality, an abnormal F/B ratio is widely recognized as a marker of gut microbiota dysbiosis.^26^

A related question is whether the microbiota differences reported above reflect the injection procedure itself rather than the endometriotic disease state. Control mice in this model underwent the same intraperitoneal injection and estradiol benzoate priming regimen as EM mice, receiving sterile PBS in place of the uterine tissue suspension: this design controls for the acute procedural stress and mechanical peritoneal irritation common to both groups. The compositional shifts detected at day 42 therefore reflect the sustained presence of ectopic lesions and the chronic peritoneal inflammation they sustain rather than the injection procedure per se, though a residual contribution from the initial immune response to the allogeneic implant is addressed further later in discussion. Spearman correlation analysis identified *g_Muribaculum, g_Ruminococcus*, and related genera as positively correlated with peritoneal LysoPC(16:0), and FMT experiments demonstrated that EM-associated microbiota causally elevates LysoPC(16:0) and promotes peritoneal M2 accumulation. The specific bacterial strains responsible for LysoPC(16:0) biosynthesis were not isolated in the present study, as the candidate genera include obligate anaerobes whose cultivation requires dedicated infrastructure not available here. FMT-based causal inference is an accepted intermediate step in establishing microbiota-metabolite relationships and has been employed in comparable studies of gut-derived lipid mediators.^27^ Future work employing single-strain gnotobiotic colonization will be required to identify the “LysoPC(16:0)-producing functional bacteria” at the species level. It is also worth noting that LysoPC(16:0) was selected as the primary effector in this study because it showed the most pronounced elevation in the peritoneal metabolome and may engage PPARγ signaling. Other co-elevated metabolites in the glycerophospholipid and linoleic acid pathways identified by KEGG analysis may contribute in concert with LysoPC(16:0), and their interactions represent a direction for future metabolite-perturbation studies.

The association between LysoPC(16:0) and immune regulation has been reported in acute liver failure and in autoimmune conditions including rheumatoid arthritis and systemic lupus erythematosus,^16^^,^^17^^,^^28^ but its role in EM had not previously been examined. The present data show that exogenous LysoPC(16:0) supplementation significantly increases ectopic lesion weight and peritoneal M2 macrophage proportions, whereas inhibiting its endogenous production with the PLA2 inhibitor AACOCF3 has the opposite effect. PLA2 has previously been shown to be a principal enzymatic source of LysoPC(16:0) generation,^29^ and the present findings further validate the importance of this biosynthetic pathway in EM pathogenesis. LysoPC(16:0), characterized by a 16-carbon palmitoyl chain and a phosphocholine head group, may engage PPARγ,^19^ a nuclear receptor that coordinates transcriptional programs governing alternative macrophage activation.^30^ In this study, the PPARγ antagonist GW9662 consistently reversed LysoPC(16:0)-induced CD206+ polarization both *in vitro* in BMDMs and *in vivo* in C57BL/6N mice. These findings were reproduced in BALB/c mice using a four-group design, confirming that the LysoPC(16:0)-PPARγ-M2 axis is not strain-specific but operates across genetically distinct backgrounds. As a central regulator of the EM lesion microenvironment, M2 macrophages suppress local immune clearance by secreting anti-inflammatory factors such as IL-10 and TGF-β, and simultaneously promote angiogenesis and fibrosis, thereby providing nutritional support for ectopic lesions.^7^^,^^31^

A key mechanistic advance of the present study is the identification of IL-10 as the downstream effector through which LysoPC(16:0)-polarized M2 macrophages promote lesion progression. IL-10 is a canonical cytokine of alternatively activated macrophages^32^ and has previously been shown to be elevated in the peritoneal fluid of patients with EM,^33^ where it suppresses immune clearance of ectopic implants and promotes lesion growth.^34^ What the present study adds is the specific upstream driver: RT-qPCR demonstrated that LysoPC(16:0) significantly upregulates *Il10* transcription in IL-4-primed BMDMs in a PPARγ-dependent manner, placing IL-10 production within the LysoPC(16:0)-PPARγ-M2 cascade. The functional importance of IL-10 was then confirmed *in vivo*: neutralizing IL-10 in LysoPC(16:0)-supplemented EM mice significantly reduced lesion weight. The clodronate liposome depletion experiment formally established that peritoneal macrophages are causally required for these effects: removing them pharmacologically abolished the pro-endometriotic effect of LysoPC(16:0) even when the metabolite was continuously supplied, consistent with earlier work demonstrating that macrophage depletion attenuates endometriotic lesion establishment and growth in mouse models.^7^^,^^35^

To provide preliminary, cell-based evidence relevant to human EM, two complementary human models were employed. THP-1-derived macrophages recapitulated the PPARγ-dependent CD206+ polarization induced by LysoPC(16:0) observed in murine BMDMs, consistent with established protocols for M2 polarization of this cell line using IL-4/IL-13 co-stimulation. Conditioned medium from LysoPC(16:0)-stimulated THP-1 macrophages then promoted migration of primary ESCs isolated from ectopic lesions of five patients with surgically confirmed EM, and this effect was consistently attenuated by GW9662 or IL-10 neutralization across all patient samples. Abnormal endometrial stromal cell migration and invasion are recognized as pivotal steps in ectopic lesion formation,^36^ and M2 macrophage-derived cytokines have been implicated in this process in both EM and related conditions such as adenomyosis.^37^^,^^38^ The consistency of the conditioned medium effect across five independent patient specimens, each tested under all six conditions in parallel, provides preliminary evidence that the LysoPC(16:0)-PPARγ-M2-IL-10 axis identified in murine models is also operative in human cells; whether this holds across the broader clinical spectrum of EM will require validation in larger patient cohorts. Co-culturing LysoPC(16:0)-stimulated BMDMs with MESCs enhanced MESC migration *in vitro*, further supporting the paracrine hypothesis. Additionally, LysoPC(16:0)-stimulated BMDMs exhibited enhanced phagocytic activity in a microsphere assay, corroborating prior reports that LysoPC promotes macrophage phagocytic function.^18^^,^^39^ In contrast, some studies have reported reduced phagocytic activity in M2 macrophages within established EM lesions,^40^ a discrepancy that likely reflects the context-dependence of macrophage phagocytic programming and the distinct stimulation conditions employed across studies.

### Limitations of the study

Several aspects of the experimental design place practical limits on these conclusions. The bacterial taxa ultimately responsible for LysoPC(16:0) production were not isolated at the species level; several candidate genera identified here are obligate anaerobes, and confirming a direct strain-metabolite relationship will require single-strain gnotobiotic colonization. The link between LysoPC(16:0) and PPARγ was established pharmacologically with the antagonist GW9662, consistent with earlier reports that structurally related lysophospholipids can act as PPARγ ligands^19^; direct biophysical evidence of binding, such as co-crystallography or surface plasmon resonance, was not obtained and would help define this interaction at the molecular level. Likewise, IL-10 was identified as the principal downstream effector, but TGF-β and other M2-associated cytokines were not systematically profiled, and how IL-10 signaling drives ESC migration—whether through JAK-STAT3, PI3K-AKT, or another route—remains to be worked out. Although control mice received an injection procedure and estradiol priming regimen identical to that of EM mice, differing only in the absence of uterine tissue, a residual contribution from the initial immune response to the allogeneic implant, as distinct from the chronic inflammation sustained by established lesions, cannot be fully excluded from the microbiota differences observed at day 42; sampling at additional time points after model establishment would help separate these two contributions.

The translational component of the study is similarly bounded by scale. Human validation relied on a monocyte-derived cell line and primary ESCs from five patients with surgically confirmed EM; although each specimen was tested in parallel across all six conditions, a cohort of this size cannot capture the clinical heterogeneity of EM across disease stage, lesion subtype, or symptom severity, and these findings are best read as exploratory, cell-based evidence rather than clinical confirmation. Establishing the clinical relevance of this axis will require larger, prospective, multi-center cohorts that relate peritoneal or circulating LysoPC(16:0) levels to disease stage and outcome. Finally, other metabolites co-elevated in the glycerophospholipid and linoleic acid pathways identified by KEGG analysis may act alongside LysoPC(16:0), and possible synergy or antagonism between them was not examined here.

Despite these limitations, the findings carry potential clinical relevance that warrants further exploration. Elevated peritoneal LysoPC(16:0) and altered microbiota composition could be evaluated as candidate biomarkers for EM in future clinical studies. Therapeutically, the PLA2–LysoPC(16:0)-PPARγ-IL-10 cascade offers multiple candidate intervention points for preclinical exploration. In conclusion, this study provides preclinical mechanistic evidence that the gut microbiota metabolite LysoPC(16:0) promotes EM progression by activating PPARγ to drive M2 macrophage polarization and IL-10 secretion, with exploratory support from human macrophages and patient-derived primary ESCs. These results nominate the “gut microbiota–LysoPC(16:0)–PPARγ–M2–IL-10” axis as a mechanistic framework for EM pathogenesis and a candidate therapeutic target for future clinical investigation.

## Resource availability

### Lead contact

Further information and requests for resources and reagents should be directed to and will be fulfilled by the lead contact, Guoyun Wang (wangguoy@sdu.edu.cn).

### Materials availability

This study did not generate new unique reagents.

### Data and code availability

• The raw sequence data reported in this paper have been deposited in the Genome Sequence Archive (Genomics, Proteomics & Bioinformatics 2025) in National Genomics Data Center (Nucleic Acids Res 2025), China National Center for Bioinformation/Beijing Institute of Genomics, Chinese Academy of Sciences^41^^,^^42^ (GSA: CRA034241) that are publicly accessible at https://ngdc.cncb.ac.cn/gsa/browse/CRA034241.
• The metabolomics data have been deposited in the Open Archive for Miscellaneous Data (OMIX: OMIX013359, OMIX013361; the latter following fecal microbiota transplantation). These data are publicly accessible at https://ngdc.cncb.ac.cn/omix/release/OMIX013359 and https://ngdc.cncb.ac.cn/omix/release/OMIX013361.
• All data in this paper will be shared by the lead contact upon request.
• This paper does not report original code.
• Any additional information required to reanalyze the data reported in this paper is available from the lead contact upon request.

## Acknowledgments

We are grateful to LC Bio Technology CO., Ltd for assisting in sequencing and bioinformatics analysis. The graphical abstract was created with Figdraw (https://www.figdraw.com). This study was funded by the 10.13039/501100012166National Key R&D Program of China (no 2023YFC2705400), the Special Foundation for Taishan Scholars (no tstp20230657), the Shandong Provincial Key R&D Program (no 2025CXPT141), the National Natural Science Foundation of China (no 82371653), and the 10.13039/501100007129Shandong Provincial Natural Science Foundation (no ZR2023MH177).

## Author contributions

M.Y. and G.W. provided the original idea and platform support. G.W. and X.J. designed the project. M.W. contributed to the experimental design and critically revised the manuscript. X.J., S.W. and Z.Z. provided invaluable guidance throughout the experiments. N.L., H.G., Y.L. and X.L. contributed to cell experiments, executing animal models, drug administration, and tissue collection. Data analysis and article writing were carried out collaboratively by N.L., X.D., Q.L. and Q.R. M.Y., X.J., and G.W. revised the article.

## Declaration of interests

The authors declare that a patent application related to this work has been filed. The details are as follows:

Application Number: 2025114516979; Applicants: Guoyun Wang, Na Li, Ming Yuan, Xueyang Jin, Xiaoyu Dong; Filing Date: October 11, 2025. No other potential conflicts of interest are declared.

## Declaration of generative AI and AI-assisted technologies in the manuscript preparation process

During the preparation of this work, the authors used DeepSeek to improve the language and readability of the manuscript. After using this tool, the authors reviewed and edited the content as needed and take full responsibility for the content of the publication.

## STAR★Methods

### Key resources table

**Table:** 

REAGENT or RESOURCE	SOURCE	IDENTIFIER
**Antibodies**		

F4/80 Monoclonal Antibody (BM8), PerCP-Cyanine5.5	Invitrogen	Cat#45–4801–82
CD206 (MMR) Monoclonal Antibody (MR6F3), PE	Invitrogen	Cat#12–2061–82
Anti-human IL-10 neutralizing antibody	BioLegend	Cat#400431
Human IgG1 isotype control	BioLegend	Cat#501427
Mouse IgG1 isotype control	MedChemExpress	Cat#HY-P99979
Anti-mouse IL-10 neutralizing antibody	MedChemExpress	Cat#HY-P990001

**Biological samples**		

Ectopic endometrial tissue (ovarian endometrioma) from patients with endometriosis	Five patients with surgically and histologically confirmed EM	N/A

**Chemicals, peptides, and recombinant proteins**		

1-Palmitoyl-2-hydroxy-*sn*-glycero-3-phosphocholine (LysoPC(16:0))	Avanti Research	Cat#855675 P
Latex beads, carboxylate-modified polystyrene, fluorescent red	Sigma-Aldrich	Cat#L3280
AACOCF3	MedChemExpress	Cat#149301–79–1
GW9662	MedChemExpress	Cat#22978–25–2
Recombinant human IL-13	PeproTech	Cat#200-13
Recombinant human IL-4	PeproTech	Cat#200-04
Empty control liposomes	Yeasen Biotechnology	Cat#40338ES10
Clodronate liposomes	Yeasen Biotechnology	Cat#40337ES10
PMA	MedChemExpress	Cat#HY-18739
TRIzol reagent	Invitrogen	Cat#15596026CN
Collagenase type I	Sigma-Aldrich	Cat#C0130
Vancomycin	Solarbio	Cat#V8050
Ampicillin	Solarbio	Cat#A8180
Metronidazole	Solarbio	Cat#M8060
Neomycin sulfate	Solarbio	Cat#N8090
Estradiol benzoate	MedChemExpress	Cat#HY-B0141
Recombinant mouse M-CSF	Thermo Fisher	Cat#AF-315-02-50UG

**Critical commercial assays**		

Hematoxylin-Eosin Stain Kit	Nanjing Jiancheng Bioengineering Institute	Cat#D006-1-1
Fecal Genome DNA Extraction Kit	BioTeke	Cat#AU46111-96
BD Cytofix/Cytoperm Kit	BD Biosciences	Cat#554714
cDNA Synthesis Kit	Accurate Biology	Cat#AG11728
SYBR Green qPCR Master Mix	Accurate Biology	Cat#AG11761

**Deposited data**		

16S rDNA sequencing raw data	This paper	GSA: CRA034241https://ngdc.cncb.ac.cn/gsa/browse/CRA034241
Peritoneal metabolomics data (control/EM groups)	This paper	OMIX: OMIX013359 https://ngdc.cncb.ac.cn/omix/release/OMIX013359
Peritoneal metabolomics data (FMT groups)	This paper	OMIX: OMIX013361 https://ngdc.cncb.ac.cn/omix/release/OMIX013361
SILVA reference database (release 138)	Quast et al.^43^	https://www.arb-silva.de/documentation/release-138/
NT-16 S database	LC-Bio Technology (in-house curated)	N/A
KEGG database	Kanehisa Laboratories	https://www.kegg.jp/
HMDB (version 4.0)	Wishart Research Group	https://hmdb.ca/

**Experimental models: Cell lines**		

THP-1	ATCC	Cat#TIB-202

**Experimental models: Organisms/strains**		

Mouse: C57BL/6N, female, 6–8 weeks	GemPharmatech	N/A
Mouse: BALB/c, female, 7–8 weeks	GemPharmatech	N/A

**Oligonucleotides**		

16 S rDNA primer 341 F: 5′-CCTACGGGNGGCWGCAG-3′	Klindworth et al.^44^	N/A
16 S rDNA primer 805 R: 5′-GACTACHVGGGTATCTAATCC-3′	Klindworth et al.^44^	N/A
*Il10* qPCR primer forward: 5′-GCTCTTACTGACTGGCATGAG-3′	This paper	N/A
*Il10* qPCR primer reverse: 5′-CGCAGCTCTAGGAGCATGTG-3′	This paper	N/A
*Gapdh* qPCR primer forward: 5′-AGGTCGGTGTGAACGGATTTG-3′	This paper	N/A
*Gapdh* qPCR primer reverse: 5′-TGTAGACCATGTAGTTGAGGTCA-3′	This paper	N/A

**Software and algorithms**		

GraphPad Prism 10	GraphPad Software	https://www.graphpad.com
FlowJo v10	FlowJo, LLC	https://www.flowjo.com
XCMS	Open source	https://www.bioconductor.org/packages/xcms
R	The R Foundation	https://www.r-project.org
QIIME2	Bolyen et al.^45^	https://qiime2.org
DADA2	Callahan et al.^46^	https://benjjneb.github.io/dada2/
LEfSe	Segata et al.^47^	https://huttenhower.sph.harvard.edu/lefse/
PICRUSt2	Douglas et al.^48^	https://github.com/picrust/picrust2
STAMP	Open source	https://kiwi.cs.dal.ca/Software/STAMP
metaX	Open source	https://github.com/wenbostar/metaX

**Other**		

Attune NxT Acoustic Focusing Cytometer	Thermo Fisher Scientific	N/A
QuantStudio 3 Real-Time PCR System	Thermo Fisher Scientific	N/A
Qubit Fluorometer	Invitrogen	N/A
Vanquish UHPLC System	Thermo Fisher Scientific	N/A
Q Exactive Mass Spectrometer	Thermo Fisher Scientific	N/A

### Experimental model and study participant details

#### Experimental animals

Female wild-type C57BL/6N mice, aged 6–8 weeks, were obtained from GemPharmatech (Nanjing, China). Female wild-type BALB/c mice aged 7–8 weeks were obtained from the same supplier. All animals were housed under specific pathogen-free (SPF) conditions in groups of up to five per cage, with a 12-h light/dark cycle, controlled temperature (22 ± 2°C) and humidity (50 ± 10%), and *ad libitum* access to standard chow and water. Drug dosing regimens were identical across strains unless otherwise specified. All procedures were approved by the Ethics Committee of Shandong First Medical University (W202311130308; approval date: November 10, 2023).

#### Human participants

Ectopic endometrial tissue (ovarian endometrioma) was obtained from five patients with surgically and histologically confirmed endometriosis (rAFS stages III–IV) undergoing laparoscopic surgery. Written informed consent was obtained from all participants prior to enrollment. The study was approved by the Ethics Committee for Human Biomedical Research of Shandong Provincial Hospital (approval no. SWYX:NO.2024–133) and conducted in accordance with the Declaration of Helsinki. Patients who had received hormonal therapy within three months before surgery or who had concurrent autoimmune, malignant, or active infectious conditions were excluded. All five participants were female. Gender identity was not separately collected. Their ages at the time of surgery ranged from 26 to 37 years (median 33 years).

#### Cell lines

THP-1 cells (ATCC, TIB-202), a human monocytic cell line, were maintained in RPMI-1640 supplemented with 10% FBS and 0.05 mM β-mercaptoethanol at 37°C in 5% CO2. Cell identity was authenticated by short tandem repeat (STR) profiling (21 loci plus Amelogenin) performed by Shanghai Biowing Applied Biotechnology Co., Ltd. (Shanghai, China) in August 2026; the STR profile matched the reference THP-1 profile in the ExPASy Cellosaurus database (accession CVCL_0006) with a match score of 0.93, consistent with authentic THP-1 identity per ATCC authentication standards (ANSI/ATCC ASN-0002-2022), and no evidence of cross-contamination (multi-allele signals) was detected. Cells were confirmed negative for mycoplasma contamination by fluorescence-based quantitative PCR testing performed by the same facility in July 2026.

### Method details

#### Construction of the mouse EM model

Donor mice received subcutaneous injections of 3 μg estradiol benzoate 7 and 2 days before model establishment; recipient mice received the same priming dose on day 5 before establishment to synchronize estrous cycles. On the day of model establishment, donor mice were anesthetized with CO_2_ and euthanized. Uteri were excised under aseptic conditions, minced into fragments, and resuspended in PBS. Each recipient mouse received an intraperitoneal injection of 200 μL of this suspension prepared from two donors, together with an additional subcutaneous dose of 3 μg estradiol benzoate. Mice were monitored daily after the procedure. Recipient mice were randomly assigned to receive an intraperitoneal injection of either the uterine tissue suspension (EM group) or an equal volume of sterile PBS (control group), with identical estradiol benzoate priming, injection timing, and post-procedure monitoring applied to both groups.

#### Vaginal cytology and estrous stage determination

Vaginal cytology was performed on days 42–45 after model establishment to confirm estrous stage. The vaginal lumen was gently flushed with 20 μL of sterile PBS, and the lavage fluid was smeared onto a glass slide and the smear was air-dried at room temperature. Slides were fixed in 95% ethanol for 10 min, stained with hematoxylin for 10 min, differentiated in 1% HCl-ethanol for 10 s, treated with bluing solution for 15 s, and counterstained with eosin for 5 min. Mice were confirmed to be in metestrus by the co-presence of keratinized epithelial cells, nucleated epithelial cells, and leukocytes in the same microscopic field.

#### Fecal and peritoneal lavage fluid collection

Mice confirmed to be in metestrus were individually housed in SPF cages without bedding. Fecal pellets were collected directly, snap-frozen in liquid nitrogen, and stored at −80°C until analysis. Mice were then euthanized by CO_2_ inhalation, and the peritoneal cavity was rinsed with 2 mL of sterile PBS under aseptic conditions. The lavage fluid was centrifuged at 1,000 rpm for 10 min followed by 2,500 rpm for 10 min; the supernatant was frozen in liquid nitrogen for 4 h and transferred to −80°C for metabolomic analysis.

#### 16 S rDNA sequencing

Fecal microbial DNA was extracted using a Fecal Genome DNA Extraction Kit (BioTeke, Beijing, China; Cat# AU46111-96) according to the manufacturer’s instructions and quantified using a Qubit fluorometer (Invitrogen). The V3–V4 hypervariable region was amplified using universal primers 341 F (5′-CCTACGGGNGGCWGCAG-3′) and 805 R (5′-GACTACHVGGGTATCTAATCC-3′)^44^ under the following cycling conditions: initial denaturation at 98°C for 30 s; 32 cycles of 98°C for 10 s, 54°C for 30 s, and 72°C for 45 s; final extension at 72°C for 10 min. Amplicons were purified using AMPure XT Beads (Beckman Coulter Genomics) and sequenced on an Illumina NovaSeq 6000 platform (PE250) by LC-Bio Technology (Hangzhou, China).

#### Bioinformatics analysis

Raw paired-end reads were merged and quality-filtered to obtain clean reads, which were denoised into amplicon sequence variants (ASVs) using DADA2 within the QIIME2 pipeline.^45^^,^^46^ Taxonomic assignment was performed against the SILVA reference database (release 138) and the NT-16 S database. Alpha diversity indices (Chao1, Shannon, Pielou’s evenness, Good’s coverage) were calculated in QIIME2 and compared using the Wilcoxon rank-sum test. Beta diversity was assessed using unweighted UniFrac distance; group separation was visualized by principal coordinates analysis (PCoA) and unweighted pair group method with arithmetic mean (UPGMA) hierarchical clustering, and tested for significance using analysis of similarities (ANOSIM). Differentially abundant taxa were identified by linear discriminant analysis effect size (LEfSe),^47^ combining the Kruskal-Wallis rank-sum test, pairwise Wilcoxon rank-sum test, and linear discriminant analysis (LDA). Indicator taxa associated with each group were identified by indicator value (IndVal) analysis among the top 30 genera by relative abundance. Functional potential was predicted from 16 S rDNA data using PICRUSt2 based on the KEGG database,^48^ and between-group functional differences were assessed using STAMP with a two-sided Wilcoxon rank-sum test.

#### Non-targeted metabolomics

Peritoneal lavage samples were processed by Lianchuan Biotechnology (China). Each 100-μL sample was mixed with 400 μL of extraction solution (methanol:acetonitrile, 1:1 v/v) containing deuterated internal standards, vortexed for 30 s, sonicated for 10 min at 4°C, and incubated at −40°C for 1 h to precipitate proteins. Samples were centrifuged at 12,000 rpm at 4°C for 15 min, and supernatants were transferred to fresh glass vials. Analysis was performed on a Vanquish UHPLC system coupled to a Q Exactive mass spectrometer (Thermo Fisher Scientific). Raw data were processed using XCMS and R software; metabolite annotation referenced the KEGG and HMDB databases.

#### Fecal microbiota transplantation

Following one week of acclimatization, mice received intragastric administration of an antibiotic cocktail (ABX; vancomycin 100 mg/kg, ampicillin 200 mg/kg, metronidazole 200 mg/kg, neomycin sulfate 200 mg/kg; Solarbio, Beijing) daily for seven days to achieve pseudo-germ-free status.^49^ EM models were established after completion of antibiotic treatment. Beginning on day 1 after model establishment and repeated every three days, mice were gavaged with 200 μL of sterile PBS (FMT-PBS), fecal suspension from control mice (FMT-NC), or fecal suspension from EM mice (FMT-EM). Fecal suspensions were prepared by resuspending 200 mg of feces in 2 mL of sterile PBS, vortexing for 5 min, and centrifuging at 800 rpm for 5 min; the supernatant was collected and administered immediately.^50^

#### Metabolite and pharmacological interventions

Beginning on the day of model establishment, mice received daily intraperitoneal injections for two weeks. In the three-group C57BL/6N experiment (Figure 5), mice were assigned to receive AACOCF3 (10 mg/kg in DMSO), LysoPC(16:0) (20 μM in chloroform:PBS, 100 μL per injection), or the corresponding vehicle. In the three-group PPARγ blockade experiment (Figure 6), mice received LysoPC(16:0) (as above), LysoPC(16:0) + GW9662 (10 mg/kg, administered 1 h before LysoPC(16:0)), or vehicle. The four-group BALB/c experiment (Figure S1) used the same dosing regimen with all four treatment arms conducted in a single experiment.

#### Macrophage depletion with clodronate liposomes

To deplete peritoneal macrophages, C57BL/6N EM mice receiving daily LysoPC(16:0) injections were co-administered clodronate liposomes (Yeasen Biotechnology, Shanghai, China; Cat# 40337ES10) or empty control liposomes (Cat# 40338ES10) by intraperitoneal injection beginning on day 1 post-model establishment and repeated every 3 days, at the concentration specified in the manufacturer’s instructions. LysoPC(16:0) was administered daily by intraperitoneal injection (100 μL per injection) as described above. Mice were euthanized at day 14; lesion photographs, lesion weights, and peritoneal F4/80^+^ cell proportions were recorded.

#### IL-10 neutralization *in vivo*

C57BL/6N EM mice receiving daily LysoPC(16:0) injections were co-administered an anti-mouse IL-10 neutralizing antibody (MedChemExpress, Cat# HY-P990001; 200 μg per mouse) or an isotype control antibody (Cat# HY-P99979; 200 μg per mouse) by intraperitoneal injection every three days for two weeks. LysoPC(16:0) was injected daily (100 μL per injection) as described above. Lesion photographs and lesion weights were assessed at the two-week endpoint.

#### Bone marrow-derived macrophage isolation and stimulation

Bone marrow cells were flushed from the femurs and tibias of 8-week-old C57BL/6N mice, filtered through a 70-μm cell strainer, and cultured in RPMI-1640 supplemented with 10% heat-inactivated FBS and 20 ng/mL recombinant mouse M-CSF for seven days to generate BMDMs. For polarization experiments, cells were stimulated for 24 h under the following conditions: NC (vehicle), IL-4 (20 ng/mL), IL-4 + LysoPC(16:0) (50 μM), or IL-4 + LysoPC(16:0) + GW9662 (10 μM; added 4 h before IL-4 and LysoPC(16:0)). For phagocytosis assays, stimulated BMDMs were incubated with PE-conjugated carboxylate-modified latex beads (Sigma-Aldrich, Cat# L3280) for 2 h at 37°C, and uptake was quantified by flow cytometry.

#### RT-qPCR

Total RNA was extracted from BMDMs using TRIzol reagent (Invitrogen) according to the manufacturer’s instructions. Reverse transcription was performed using a standard cDNA synthesis kit. Quantitative PCR was carried out using SYBR Green qPCR Master Mix (Accurate Biology, Cat# AG11761) on a QuantStudio 3 real-time PCR system (Thermo Fisher Scientific), following the manufacturer’s recommended cycling program. Primer sequences for *Il10* were: forward 5′-GCTCTTACTGACTGGCATGAG-3′, reverse 5′-CGCAGCTCTAGGAGCATGTG-3′. *Gapdh* was used as the reference gene. Relative expression levels were calculated using the 2−ΔΔCt method.

#### Mouse endometrial stromal cell isolation and transwell migration assay

Mouse endometrial stromal cells (MESCs) were isolated from uterine tissue by enzymatic digestion and cultured to passage 3 before use. For the co-culture migration assay, 2 × 10ˆ5 MESCs were seeded in the upper chamber of 8-μm pore Transwell inserts, and 1 × 10ˆ6 differentially stimulated BMDMs were seeded in the lower chamber. After 24 h of co-culture, non-migrated cells were removed from the upper surface of the membrane, and migrated cells on the lower surface were fixed with 4% paraformaldehyde, stained with crystal violet, and counted from five randomly selected fields per insert under light microscopy.

#### Flow cytometry of peritoneal macrophages

Peritoneal lavage cells were stained with PerCP-Cy5.5-conjugated anti-F4/80 (clone BM8; Invitrogen) for 30 min at 4°C. Cells were then fixed and permeabilized using a BD Cytofix/Cytoperm Kit (BD Biosciences) for 30 min, followed by intracellular staining with PE-conjugated anti-CD206 (clone MR6F3; Invitrogen) for 60 min at 4°C in the dark. Data were acquired on an Attune NxT Acoustic Focusing Cytometer (Thermo Fisher Scientific) and analyzed using FlowJo v10 software.

#### THP-1 macrophage differentiation and stimulation

Differentiation into macrophages was induced by treatment with PMA (10 ng/mL; MedChemExpress) for 24 h, followed by a 24-h rest period in fresh medium without PMA.^51^ Differentiated cells were then stimulated for 24 h under the following conditions: NC (vehicle), IL-4 (20 ng/mL; PeproTech, Cat# 200-04) + IL-13 (20 ng/mL; PeproTech, Cat# 200-13), IL-4/IL-13 + LysoPC(16:0) (50 μM), or IL-4/IL-13 + LysoPC(16:0) + GW9662 (10 μM; pre-treated 4 h before co-stimulation). For IL-10 neutralization conditions, anti-human IL-10 antibody (BioLegend, Cat# 400431; 10 μg/mL) or human IgG1 isotype control (BioLegend, Cat# 501427; 10 μg/mL) was added simultaneously with LysoPC(16:0). CD206+ polarization was assessed by flow cytometry as described above. Conditioned medium was collected after 24 h stimulation, cleared by centrifugation at 300 × g for 5 min to remove cell debris, and used immediately for ESC migration assays.

#### Human primary ESC isolation and migration assay

Ovarian endometrioma tissue obtained at surgery was rinsed with sterile PBS and minced into fragments under aseptic conditions. Tissue fragments were digested in DMEM/F12 containing collagenase type I (1 mg/mL; Sigma-Aldrich, Cat# C0130) at 37°C; the suspension was triturated by pipetting every 15 min until no visible tissue fragments remained. The digestion was terminated by addition of FBS, and the cell suspension was centrifuged at 400 × g for 5 min. The pellet was resuspended in red blood cell lysis buffer and incubated for 8 min at room temperature, then centrifuged again at 400 × g for 5 min. The resulting cell pellet was resuspended in DMEM/F12 supplemented with 10% FBS and 1% penicillin-streptomycin and plated for culture. Cells were passaged at approximately 80% confluency and used at passage 2–3 for all experiments.

For the migration assay, 2 × 10ˆ5 ESCs in serum-free medium were seeded in the upper chamber of 8-μm pore Transwell inserts. Conditioned medium from THP-1 macrophages stimulated under each of the six conditions (NC, IL-4/IL-13, IL-4/IL-13 + LysoPC(16:0), IL-4/IL-13 + LysoPC(16:0) + GW9662, IL-4/IL-13 + LysoPC(16:0) + anti-IL-10, IL-4/IL-13 + LysoPC(16:0) + isotype control) was placed in the lower chamber. After 24 h, non-migrated cells were removed from the upper membrane surface, and migrated cells were fixed, stained with crystal violet, and counted from five randomly selected fields per insert. Each patient’s cells were tested across all six conditions in the same experimental session.

### Quantification and statistical analysis

Data are presented as mean ± SD or mean ± SEM, as specified in the corresponding figure legends. Comparisons between two groups were performed using unpaired two-tailed Student’s t-tests, unless otherwise specified in the corresponding figure legends. For comparisons among three or more groups, ordinary one-way ANOVA or, when variances were unequal, Brown-Forsythe and Welch ANOVA was used, each with the post hoc multiple-comparison test specified in the corresponding figure legends; non-parametric alternatives were applied where indicated. Spearman’s rank correlation was used for microbiota–metabolite association analysis. Statistical analyses of the 16S rDNA sequencing data were performed within the QIIME2, LEfSe, and STAMP pipelines, and metabolomics data were processed using XCMS and R, as described above; all other statistical analyses were performed using GraphPad Prism 10. A *p*-value of less than 0.05 was considered statistically significant. *In vivo* group sizes (*n* = 6 per group) were consistent with sample sizes commonly reported in published murine models of endometriosis, including studies employing comparable fecal microbiota transplantation or lesion-comparison designs.^52^^,^^53^^,^^54^ Sample sizes for individual experiments are specified in the corresponding figure legends.

### Additional resources

Not applicable.

## Supplemental information

Document S1. Figures S1 and S2

Data S1. Raw flow cytometry data files and the corresponding FlowJo analysis workspaces underlying the macrophage quantifications shown in Figures 4C and 7D, related to Figures 4 and 7 The archive contains two folders: figure4C, with the.fcs files and the FlowJo workspace used for Figure 4C; and Figure7D, with the.fcs files, the FlowJo workspace, and a sample correspondence table used for Figure 7D.
