# Supplementary material for: Intestinal microbiota-associated LysoPC(16:0) promotes endometriosis via PPARγ-mediated M2 macrophage polarization and IL-10 secretion
Source: iScience. 2026 Oct 15;29(11):117917. doi: 10.1016/j.isci.2026.117917 (PMC13691365; doi:10.1016/j.isci.2026.117917)
Supplement: Supplementary file 1 [file mmc1.pdf]

iScience, Volume *29*

Supplemental information

Intestinal microbiota-associated LysoPC(16:0) promotes endometriosis via PPARγ-mediated M2 macrophage polarization and IL-10 secretion

Na Li, Ming Yuan, Xueyang Jin, Xue Jiao, Shuang Wang, Zhaoyang Zhong, Hongwei Guan, Xu Le, Qianhui Ren, Yu Liu, Xiaoyu Dong, Qiuju Li, Mei Wang, and Guoyun Wang

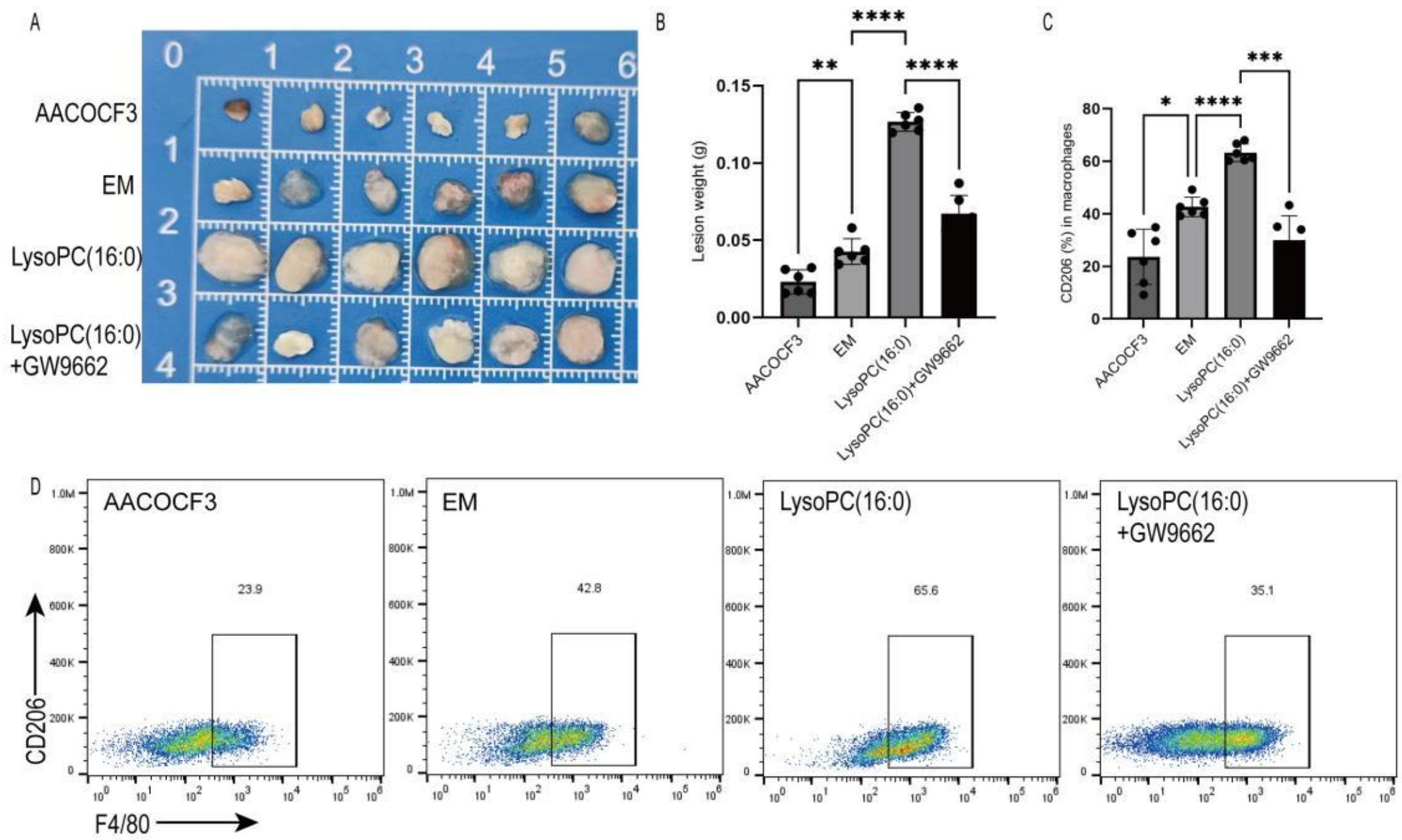

Figure S1. Cross-strain validation in BALB/c mice, related to Figure 6.

(A) Representative lesion photographs, photographed alongside a graduated scale board (cm/mm divisions). (B) Lesion weight; ordinary one-way ANOVA with Šídák's multiple comparisons test among sequential groups. (C) Proportion of peritoneal CD206+ macrophages; Brown-Forsythe and Welch ANOVA with Dunnett's T3 multiple comparisons test. (D) Representative flow cytometry plots showing the proportion of CD206+ macrophages in peritoneal lavage fluid from BALB/c EM mice treated with AACOCF3, EM vehicle, LysoPC(16:0), or LysoPC(16:0) + GW9662. n = 6 mice per group; *p < 0.05, **p < 0.01, ***p < 0.001, ****p < 0.0001. Data are represented as mean ± SD.

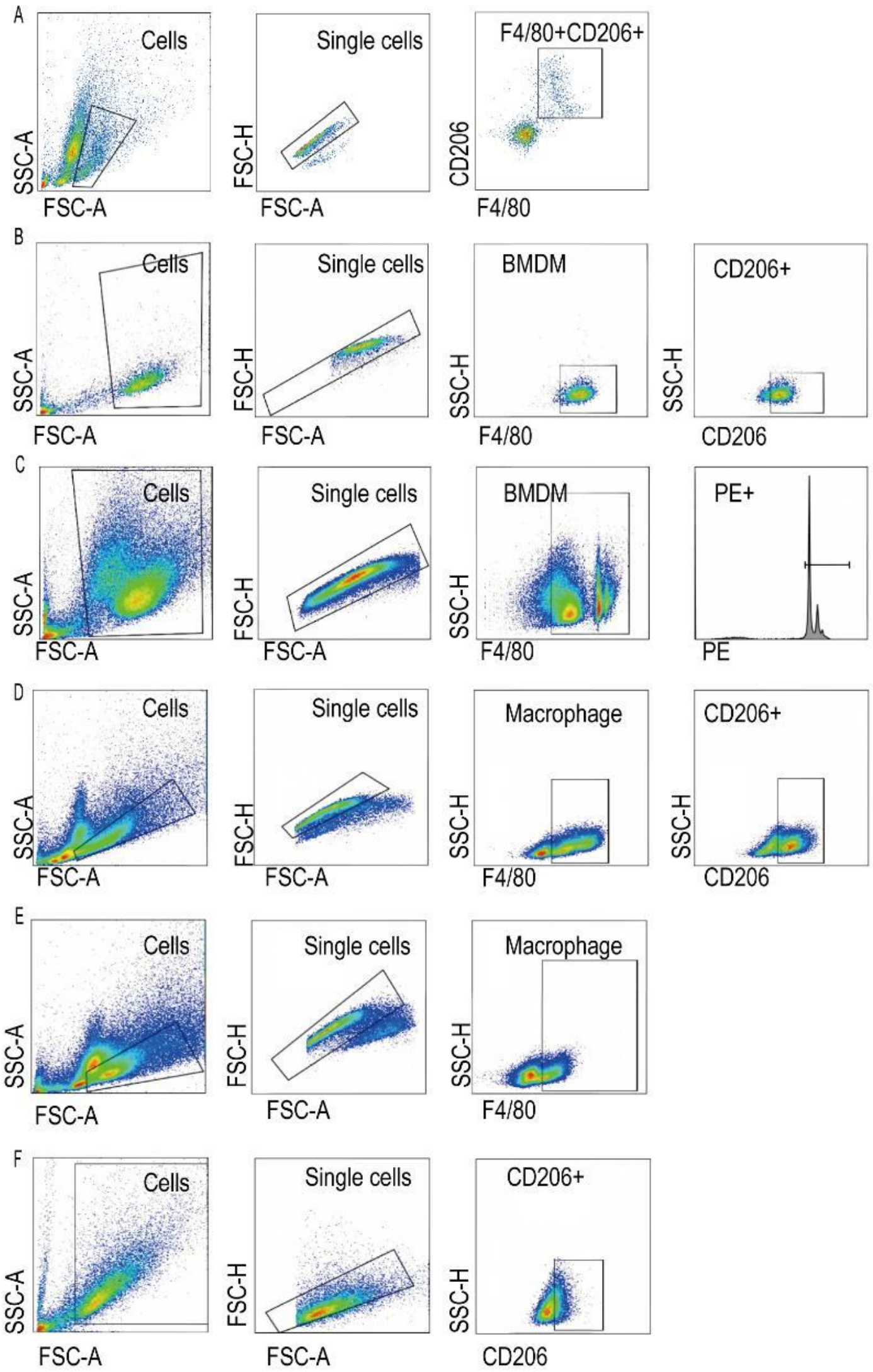

Figure S2. Representative flow cytometry gating strategies used throughout this study, related to Figures 3–8 and S1.

(A) Gating strategy using a combined F4/80+CD206+ gate (Cells → Single cells → F4/80+CD206+). Related to Figures 3H, 4C, 5F, and S1D. (B) Gating strategy using sequential F4/80+ then CD206+ gates for BMDMs in vitro (Cells → Single cells → F4/80+ → CD206+). Related to Figures 4A, 4B, and 6A. (C) Gating strategy for the phagocytosis assay, showing PE-conjugated microsphere uptake (Cells → Single cells → F4/80+ → PE+). Related to Figure 4G. (D) Gating strategy for peritoneal macrophages in the in vivo PPARγ validation cohort (Cells → Single cells → F4/80+ → CD206+). Related to Figure 6H. (E) Gating strategy for peritoneal F4/80+ macrophages in the clodronate depletion experiment (Cells → Single cells → F4/80+). Related to Figure 7D. (F) Gating strategy for THP-1-derived macrophages (Cells → Single cells → CD206+). Related to Figures 8A and 8B. Gates were set based on unstained and/or fluorescence-minus-one controls and applied uniformly across all treatment groups within each panel.
