# Supplementary material for: Intestinal microbiota-associated LysoPC(16:0) promotes endometriosis via PPARγ-mediated M2 macrophage polarization and IL-10 secretion
Source: iScience. 2026 Oct 15;29(11):117917. doi: 10.1016/j.isci.2026.117917 (PMC13691365; doi:10.1016/j.isci.2026.117917)
Supplement: Supplementary file 2 [file mmc2.zip › Data_S1/figure7D/Figure7D_Sample_Correspondence_Table_EN.docx]

# Figure 7D — Source FCS File and Sample Correspondence Table

Manuscript: ISCIENCE-D-25-18058R5

Workspace file: figure7D-FCS.wsp (FlowJo 10.9.0)

Gating hierarchy: All events → Singlets → F4/80+ (PerCP-Cy5.5) — proportion shown is %F4/80+ of singlets, matching the Figure 7C/7D quantification.

## Clodronate liposome (CL) and empty liposome (EL) groups, n = 6 per group

| **Original filename** | **Group** | **F4/80+ % of singlets** | **Role in Figure 7** |
| --- | --- | --- | --- |
| jushiqingchu(1).fcs | CL | 6.88% | Quantification replicate (Fig 7C) |
| jushiqingchu(2).fcs | CL | 6.17% | Quantification replicate (Fig 7C) |
| jushiqingchu(3).fcs | CL | 4.10% | Quantification replicate (Fig 7C) |
| jushiqingchu(4).fcs | CL | 6.10% | Quantification replicate (Fig 7C) |
| jushiqingchu(6).fcs | CL | 5.38% | Quantification replicate (Fig 7C) |
| jushiqingchu(16).fcs | CL | 5.16% | Representative flow plot (Fig 7D, left) |
| m2.fcs | EL | 27.8% | Quantification replicate (Fig 7C) |
| m3(2).fcs | EL | 34.70% | Quantification replicate (Fig 7C) |
| m3(7).fcs | EL | 37.8% | Representative flow plot (Fig 7D, right) |
| m3(8).fcs | EL | 33.2% | Quantification replicate (Fig 7C) |
| m3(9).fcs | EL | 36.0% | Quantification replicate (Fig 7C) |
| m3(12).fcs | EL | 25.2% | Quantification replicate (Fig 7C) |

## Compensation / staining controls (not part of the n = 6 quantification)

| **Original filename** | **Group** | **F4/80+ % of singlets** | **Role in Figure 7** | **Corresponding panel** |
| --- | --- | --- | --- | --- |
| cd206.fcs | Single-stain control | N/A | CD206-PE compensation control; not experimental group data | — |
| f480.fcs | Single-stain control | N/A | F4/80-PerCP-Cy5.5 compensation control; not experimental group data | — |
| inos.fcs | Single-stain control | N/A | iNOS-APC-eFluor780 compensation control; not experimental group data | — |
| yin.fcs | Negative control | N/A | Unstained/negative control; not experimental group data | — |

## Notes

1. File names reflect the cytometer acquisition software's automatic sequential numbering within the acquisition session (e.g., jushiqingchu(1)...(16)); non-sequential numbers correspond to samples from other staining panels/experimental groups acquired in the same session and are not part of this dataset.

2. Figure 7D displays representative flow cytometry plots; the values shown on the plots (CL = 5.56%, EL = 36.0%) correspond to jushiqingchu(16).fcs and m3(7).fcs among the n = 6 biological replicates per group.

3. Figure 7C reports the quantified proportion across all n = 6 biological replicates per group (unpaired Welch's t test), corresponding to all files listed above under each group.
